# Evaluation of Capacity-Building Program of District Health Managers in India: A Contextualized Theoretical Framework

**DOI:** 10.3389/fpubh.2014.00089

**Published:** 2014-07-25

**Authors:** N. S. Prashanth, Bruno Marchal, Guy Kegels, Bart Criel

**Affiliations:** ^1^Institute of Public Health, Bangalore, India; ^2^Department of Public Health, Institute of Tropical Medicine, Antwerp, Belgium

**Keywords:** capacity-building, realist evaluation, district health management, theory-driven research, health managers, health manpower, human resources for health, local health system

## Abstract

Performance of local health services managers at district level is crucial to ensure that health services are of good quality and cater to the health needs of the population in the area. In many low- and middle-income countries, health services managers are poorly equipped with public health management capacities needed for planning and managing their local health system. In the south Indian Tumkur district, a consortium of five non-governmental organizations partnered with the state government to organize a capacity-building program for health managers. The program consisted of a mix of periodic contact classes, mentoring and assignments and was spread over 30 months. In this paper, we develop a theoretical framework in the form of a refined program theory to understand *how* such a capacity-building program could bring about organizational change. A well-formulated program theory enables an understanding of how interventions could bring about improvements and an evaluation of the intervention. In the refined program theory of the intervention, we identified various factors at individual, institutional, and environmental levels that could interact with the hypothesized mechanisms of organizational change, such as staff’s perceived self-efficacy and commitment to their organizations. Based on this program theory, we formulated context–mechanism–outcome configurations that can be used to evaluate the intervention and, more specifically, to understand what worked, for whom and under what conditions. We discuss the application of program theory development in conducting a realist evaluation. Realist evaluation embraces principles of systems thinking by providing a method for understanding how elements of the system interact with one another in producing a given outcome.

## Introduction

The local health system at the district level is an important organizational unit for management of health services. In India and many other low- and middle-income countries (LMICs), doctors are usually in charge of the management of local health systems. As health managers, doctors lead a team of health workers that includes other doctors and clinical specialists, nurses and midwives, pharmacists, laboratory and other technicians, and administrators. A well-functioning local health system is able to translate its inputs (human, financial, and technical resources) into processes and outputs (health care). In addition, it should ensure that the health care provided is organized and managed in such a way that it is physically and financially accessible, equitable, of good quality, and responsive to local needs (1).

Many district health systems in LMICs do not have the capacity to allocate their financial resources equitably and manage their technical resources optimally (2). Furthermore, the health workforce is unequally distributed leading to skill mix problems (3). This affects the quality of the healthcare provided and thus the health status of the people. Moreover, in the absence of a well-functioning local health system, disease-control programs are hampered in achieving their goals, in spite of their good design. Indeed, there are instances of such programs failing or even having a harmful effect on health systems, specifically on planning, monitoring, and evaluation (4–6).

It is now generally acknowledged that strong health systems are needed, but studies and reviews on health system strengthening and more specifically on capacity-building in health (and how it improves performance) are few [Ref. (7), see Box 1]. In parallel, attention for complexity in health is growing. There have been calls for increasing scientific evaluation of complex interventions in health to improve our understanding of *what works for whom and under what conditions* (8–12).

Box 1 **Capacity-building through training programs: what do we know?**

As an alternative to systematic reviews, realist synthesis is emerging as a way to expand our knowledge base, especially when attempting to answer context-sensitive and policy-relevant questions (13, 14). A realist synthesis begins with the question: “what works for whom under what circumstances, how and why?” (13, 14). A recently published realist synthesis of human resource management (HRM) interventions in LMICs could identify only 48 scientific articles, of which 21 were related to capacity-building through training programs (7). In these 21 articles, the synthesis reports that “… the mechanisms through which training produced changes were researched in (only) three studies.” The report found that “… improvement of health worker performance was triggered by three distinct mechanisms: improved knowledge and skills, critical awareness on the functioning of health services, and being empowered to implement change.”

We have developed a realist evaluation study to understand how capacity-building of health managers translates into improved performance with respect to their planning and supervision in Tumkur district of Karnataka state in southern India (15). A realist evaluation aims to produce a context-specific understanding of the mechanisms through which a given outcome is produced [Ref. (16), see Box 2]. The first step toward understanding how an intervention produces the expected outcomes is to understand the intervention and all its elements, and gain insight into how it interacts with the actors and the various components of the recipient health system. Capacity-building programs of health workers are embedded in the existing organizational and socio-political context of the area where they are implemented. Hence, in addition to the program inputs, the relationships between the actors in the system and the interaction between the program elements and the recipient health system, affect the program outcomes.

Box 2 **Realist evaluation begins by asking (about programs or policies), what works, how, in which conditions, and for whom (17)**

The outcome of a realist evaluation is an empirically tested and context-specific explanation for why the program or policy worked for some and not for others. The evaluators begin by refining the initial program theory of the program (or policy); the initial program theory is based on the assumptions made by the designers/implementers on why the inputs of the program will bring about the desired output. The evaluator seeks to refine this initial program theory in order to understand the local contextual conditions that influence the outcome, as well as the possible causal mechanisms that could have resulted in the outcome. Data are collected and analyzed using the conjectural context–mechanism–outcome (CMO) configurations; configurations consisting of causal mechanisms that explain the observed outcome, in relation to specific contextual conditions that allow for these mechanisms to operate. The CMO configurations are an analytical tool consisting of testable conjectures drawn from the program theory that help in generating an explanation for *what works, for whom, and under what conditions*. The current understanding of context and mechanism in realist evaluation is summarized below (18).

*Context* – actors or other factors that occur in the setting where the intervention/policy was implemented, that occur independent of the intervention/policy, and affect the implementation of the intervention/policy.

*Mechanisms* – psychological or social explanations for human behavior that explain the interaction between social structure and individual/group agency.

**Figure 1:**
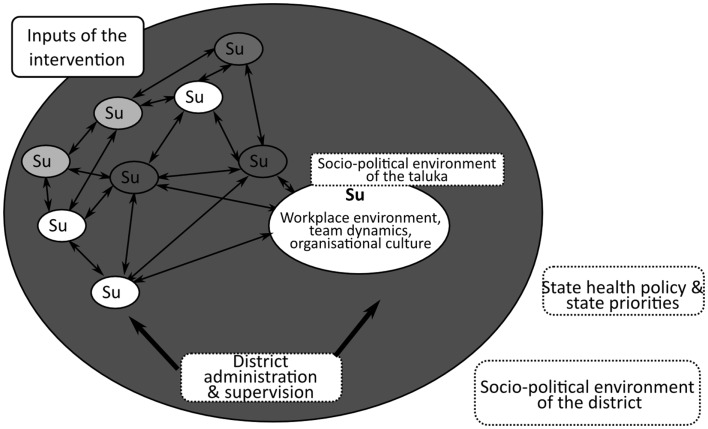
**A generic representation of an intervention and its interaction with the district health system and its various sub-units (Su)**. Sub-units could include hospitals, disease-control programs, or talukas (sub-districts). A district level intervention interacts within and among the constituent local health systems in the district as well as the policy environment of the district (22).

The frameworks used to describe and analyze health systems have evolved in response to the acknowledgment of complexity of these systems (1, 19–21). The response of providers and managers of hospitals or health centers to a given intervention will depend on the dynamic interactions between various factors operating at different levels in their system. Hence, interventions at district level could result in positive change in some people or in some institutions, while not in others. In order to understand *how* a given intervention could bring about positive organizational change, the interaction between and among the various individuals within the sub-units of the system needs to be analyzed. A systems’ approach to address complexity within a district health system requires that the relationships between the sub-units of system, and the possible ways in which they could interact and affect the *production* of the outcome, should be understood and made explicit (see Figure 1).

Recent evaluation studies have called for approaches that help unpack the black-box between an intervention’s inputs and its outcomes (23, 24). These studies are centered around the development of a program theory approach that allows for the formulation of a plausible basis for expected outcomes, drawing from the body of knowledge on the given intervention (literature), as well as building on the role of local context in shaping these outcomes.

In this paper, we progressively develop the program theory of the capacity-building intervention, which was launched in Tumkur, India in 2009. The intervention involved training of health managers on public health management topics through a mix of classroom teaching and mentoring at their workplace. The formulation of a program theory enables identification of plausible mechanisms through which organizational change could have occurred, and the contextual factors that triggered these mechanisms. In realist evaluation, the mechanisms could be understood as the agency through which the resources introduced into the system by the program produce the outcome. However, context, in the form of the appropriate external environment (or not), is critical for the manifestation of the outcome (17). A detailed description of the intervention is provided in File S1 in Supplementary Material.

## Materials and Methods

### The study setting

In India, local health systems at the district level are generally organized in three tiers. The first point of contact between people and healthcare professionals is a network of Primary Health Centers (PHC), which depend on a hospital at the sub-district level (taluka) for secondary care. A district hospital is supposed to provide tertiary specialty care. On average, a district in India has a population ranging from 500,000 to a few million people. A sub-district (called taluka in Karnataka) would generally have a few hundred thousand inhabitants. The production of healthcare outputs of hospitals and health centers is complex; it is influenced by the internal dynamics of the workforce in these organizations, by the relationships among individual health workers, as well as by their external environment (25). Environmental factors include local socio-political, governance, and policy influences from the higher levels in the system. An overview of the organization and management of health services in Karnataka state and the policy context within which the intervention operated is summarized in File S2 in Supplementary Material.

We developed the program theory in a step-wise fashion based on guidance in literature (16, 17, 26). We first summarized prior theory and research on the subject, then collected data on contextual factors that could affect the expected outcomes of the intervention, and finally formulated the implicit theory of the intervention. Based on this program theory, we eventually developed CMO configurations helping us to understand how the intervention could have worked, for whom, and under what conditions. A realist evaluation of health management interventions builds upon such CMO configurations in order to generate plausible explanations on how this intervention could have worked (24, 27).

The steps followed are described below and summarized in Table 1.

(1) Understand the intervention (initial program theory, IPT): we started with eliciting the IPT of the intervention. These are the assumptions and hypotheses of the designers of the intervention and other stakeholders. To do so, we reviewed program documents (list of documents in File S3 in Supplementary Material) to identify the implementers’ main assumptions, to understand the perceptions of the key actors and to identify potential mechanisms – if any – as identified by the implementers. Implementers are the people and organizations who designed and are in charge of the implementation of the intervention. At this stage, we were looking for assumptions of the designers on how and why the program would bring about the expected outcomes. In a second step, we interviewed 16 actors using an interview guide (File S4 in Supplementary Material): 2 program designers, 2 policymakers, 10 participants (health managers), and 2 health services staff during the early phase of the implementation of the intervention. The interviews focused on the process of planning health services, the perceived scope for change given the current decentralization process, and the possible role of the intervention in this change. The program documents and interview transcripts were imported into NVivo 10 (QSR International Pvt. Ltd., Australia). Portions of text reflecting implementation assumptions, possible contextual factors (see step 3 below), and actors’ perceptions were coded and analyzed using thematic analysis. These themes were then summarized and the IPT was gradually constructed.
(2) Literature review to identify possible mechanisms: we reviewed the published literature and identified possible steps through which capacity-building can lead to organizational change. We began the literature search on the basis of four themes highlighted in the IPT: organizational commitment, self-efficacy, workplace learning, and evaluation of training programs. We conducted the search initially on Google Scholar and PubMed; we also scanned the references and carried out citation tracking of some of the papers we had retained, to identify other key publications (14). We finally retained articles (primary research, review articles, and reports) based on our assessment of the article’s relevance to our program theory: organizational commitment (36 papers), self-efficacy (19 papers), workplace learning (6 papers), and evaluation of training programs (57 papers). The list is presented in File S5 in Supplementary Material.
(3) Identify contextual factors: we reviewed government reports and program documents related to performance of district health services (full list of documents in File S3 in Supplementary Material). We also analyzed the interview transcripts with participants of the intervention, co-workers of the participants, policymakers, and implementers to identify contextual factors that could possibly influence the actors, the implementation, and the outcomes of the intervention. Key events that affected the implementation of the capacity-building intervention were also identified from the interview transcripts and these were mapped. The interview transcripts were imported into NVivo10 (QSR International, Australia) and free coding was done to identify important factors presenting at various levels of the health system. The codes were then organized into trees and the themes emerging were summarized.
(4) Refine program theory: we integrated possible mechanisms (from step 1 and 2) and contextual factors (from step 3) into the refined program theory.
(5) Formulate CMO configurations: a framework for plausible configurations of CMO was constructed from which empirically testable hypotheses can be drawn.

**Table 1:** **Steps in building the program theory of the intervention**.

Steps	Question	Method	Outcome
Understanding the intervention	How was the intervention supposed to work? What was the response of the actors in the system vis-à-vis the assumptions of the implementers?	Review of program documents, meeting minutes, reports, and transcripts of interviews with implementers	Input–outcome logic model connecting intervention inputs to outcomes showing possible intermediate steps (Figure 2)
Review literature	What do we know (from published literature) about how such interventions could work?	Narrative review of literature on organizational commitment, self-efficacy, workplace learning, and evaluation of training programs	Synthesis of literature on capacity-building in health with a focus on the mechanisms
Identify contextual factors	What are the conditions in the district health system that affect the expected outcome?	Review of program implementation reports, observation notes and interview transcripts	Contextual factors identified
Refine program theory	How could the intervention lead to improved organizational performance?	Integrate contextual factors and mechanisms into the initial program theory	Plausible relationship between the elements of the intervention and its expected outcomes (Figure 3)
Formulate context–mechanism–outcome (CMO) configurations	What worked, for whom, and under what conditions?	Identify configurations of CMO that could be empirically tested	CMO configurations that could be empirically tested

We used the *multipolar performance framework* to analyze and discuss the refined program theory (28). This framework is an adaptation of a conceptual framework designed for analyzing the performance of healthcare organizations proposed by Sicotte et al. (29). It integrates several theories on organizational change and has been applied to explain healthcare organizations’ performance in LMICs settings (1, 28–30).

## Results

We present the results of the five steps described above, followed by a discussion of the results using the multipolar performance framework.

We first described the goal of the intervention, its rationale, the components of the intervention, the participants (and other actors involved), and the implementation timelines (File S1 in Supplementary Material). For the purpose of this study, we will focus on two of the key expected outcomes of the capacity-building program viz. improved annual action plans at district and taluka level and improved supervision practices. These were the two major outcomes that the designers sought to influence through the capacity-building program. An interim self-evaluation of the intervention by the implementers, as well as an external evaluation, highlighted planning and supervision as possible key outcomes (31, 32). We then present the literature review and the context analysis, and eventually bundle the refined program theory and the CMO configurations.

### Understanding the intervention: Initial program theory

**Figure 2:**
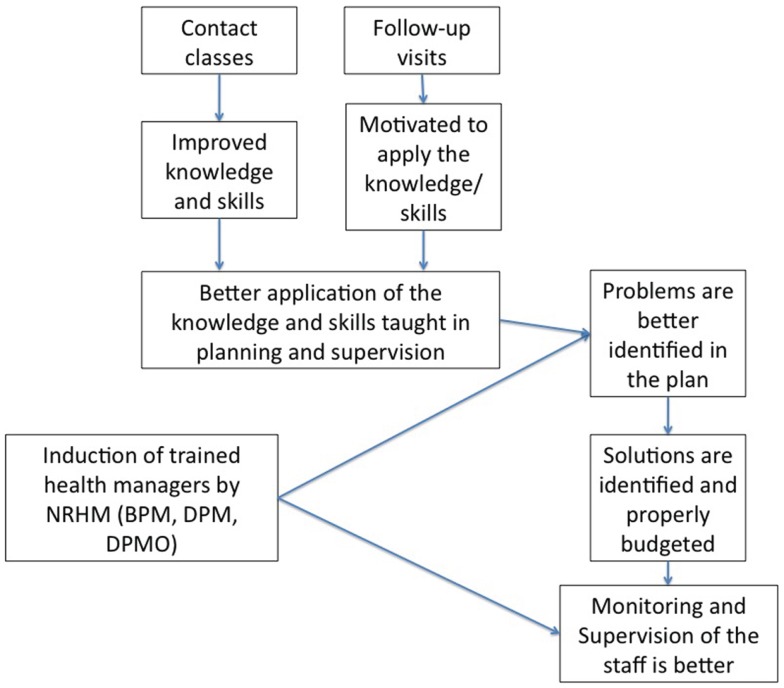
**The initial program theory of the intervention as conceived by the program designers**. Reproduced from the study protocol (15).

The IPT is readily available – see the publication of the study protocol (15) – reproduced in Figure 2. The IPT is therein schematized as a linear representation of the intervention’s inputs (contact classes and mentoring of participants) connected to the intervention’s expected outcomes (improved annual action plans and supervision practices) through a set of intermediate steps (better problem and solution identification, better monitoring and more supportive supervision). The IPT considered the posting of non-medical management professionals at district and sub-district levels [as part National Rural Health Mission (NRHM) – see below for description of the NRHM] as a significant contextual factor, expected to influence the intervention’s outcomes. Based on this IPT, the intervention could be formulated as a human resources management intervention consisting of an in-service training and mentoring program to bring about organizational change in district health management, through improved preparation and implementation of annual action plans and supportive supervision. The IPT was represented as a logic model. However, from a realist evaluation perspective, it requires further elaboration by making explicit the assumptions of the program designers on the possible intermediary steps and by taking into consideration the contextual factors that could affect the implementation of the intervention. Also, potential mechanisms of change need to be identified.

Based on documentary review and thematic analysis of the interviews with the designers of the intervention, we identified three key assumptions that *a priori* guided the design and implementation of the intervention. These assumptions have had implications on how the program was structured (e.g., role of experienced teachers visiting the participants as mentors, whom to include in the program), its content (e.g., what type of content to include and how to present them), and the implementation (e.g., focus on improving the district and taluka health system and focusing on health management teams at these levels). These assumptions are the following:

#### An attitudinal change among the participants is needed to achieve the desired results

The program implementers noted that improved public health management knowledge and skills are insufficient by themselves in bringing about change. They thought that an attitude toward creating organizational change among the participants is essential. The implementers sought to encourage or bring about such an attitudinal change by using particular styles of teaching (e.g., applying adult-learning techniques such as participatory and peer/group learning in the contact classes), letting participants identify examples of glaring gaps in existing services, and mentoring participants at their own workplaces. The mentors were either the teachers from the contact classes or experienced public health professionals. They would visit the participants at their workplace, discuss topics raised in the contact classes, and offer to demonstrate or help implement the acquired knowledge and/or skills in the participants’ workplace. The implementers described the changed attitude that they aimed for, as a *can-do* attitude. This was based on the perception of the designers that there was apathy and lack of desire to change things at the district and taluka level. The implementers assumed that the participation of experienced health professionals in mentoring visits could trigger such an attitude among the participants, and hence create an environment where the knowledge and skills would be put to effective use.

#### The program can benefit from and take into account alignment with existing policy initiatives

The Indian government’s flagship health program, the NRHM, is being implemented since 2005. The NRHM sought to bring about an architectural correction in the health system through improving financing arrangements and reforms in planning and supervision of health services. One of the reforms was decentralized planning and management of health services to the district level (33). Our analysis shows that the program implementers felt that the objectives of the capacity-building program aligned with the new resources coming through the NRHM and NRHM’s efforts at district level decentralization. They therefore included the newly created cadre of health managers at taluka and district level – the Block Program Managers (BPMs) at sub-district level and the District Program Managers (DPMs) at district level – as participants in the intervention. They also identified the new system of decentralized planning at the district level as an opportunity for the district health team to implement organizational change through improved annual action plans and better supervision practices.

#### Targeting individuals (for the capacity-building program) will produce impact through teams

The implementers identified health management teams at hospitals, talukas, and disease-control programs as the unit for change within the health services. These teams included medical doctors and administrative staff, but also the newly introduced BPMs and DPMs. The implementers (and NRHM) expected that the induction of these new cadres and the building of teams at taluka and district levels would improve local annual action plans; earlier, action plans were largely made at the state-level in a top-down fashion. The program implementers specifically targeted these enlarged management teams.

**Figure 3:**
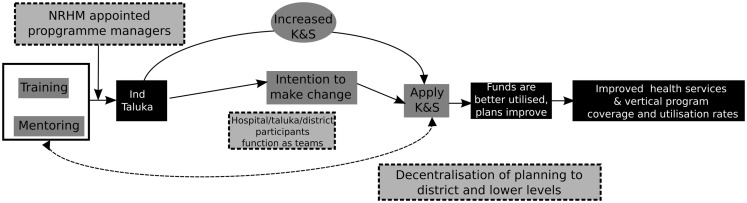
**Program theory of the intervention after incorporating the assumptions of the implementers**.

The program theory incorporating the assumptions of the implementers is represented in Figure 3.

### Review of literature: How are capacity-building interventions supposed to work?

Human resource management interventions can be important drivers of health service provision and thus good health outcomes. However, studies on HRM in health are few; they focus mainly on continuing education, supervision, payment of incentives, decentralization of HRM functions, or a combination of these (7, 34). An understanding of the mechanisms through which HRM interventions produce change in healthcare institutions is crucial for the design and delivery of such interventions in LMICs settings. However, the current evidence base for how positive organizational change could be achieved through capacity-building based HRM interventions in health services is scarce (7, 34, 35). Both systematic and realist reviews of studies in human resources for health note that the role of context in producing desirable outcomes in HRM interventions is under-explored; either contextual factors are neglected in designing effectiveness evaluations, or context descriptions are scarce, rendering the studies not amenable to realist reviews (7, 22).

**Figure 4:**
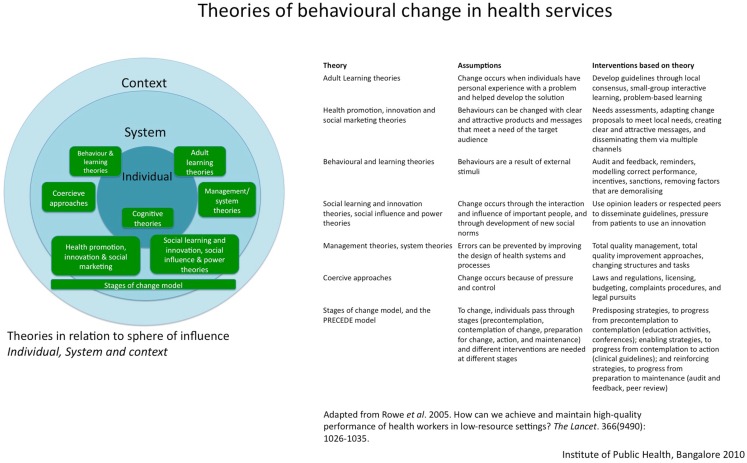
**Theories of behavioral change in health services in relation to their sphere of influence**. Based on the review of theories that explain behavioral change in health services by Rowe et al. (34).

Contrary to the evidence base on HRM in the public health literature, much has been written on the topic in management sciences, particularly in the corporate business industry (36). In a review focusing on evidence on achieving and maintaining good performance of health workers in LMICs, Rowe et al. (34) identify eight theories underlying most HRM interventions in health. These theories explain organizational improvement through change in health worker behavior and practices, which they place across several levels: the team, the institution, and the larger health system environment within which they work. The theories reviewed are summarized in Figure 4. They could be thought of as providing explanations of change seen at individual, institutional, and systems levels; however, Rowe et al. note that: “… little is known about how well the theories predict health worker practices or success of interventions.” Furthermore, most studies included in their review concern healthcare workers. Studies pertaining to local health system managers, mostly doctors in the case of Indian districts, are scarce.

Capacity-building programs inject new resources, i.e., knowledge, skills, and experiences, in organizational settings. A variety of individual, institutional, and environmental factors determine who benefits from such programs and who does not, and who applies what they learnt in terms of organizational change, and who does not. Among the frameworks proposed to evaluate the effect of training programs, the Kirkpatrick and Kirkpatrick framework is one of the most frequently used (37). The framework proposes four levels at which the training programs could be evaluated: reaction (to the training), learning (knowledge and skills), behavior (applying the new learning), and impact (changes brought in the organization). In a critical analysis of evaluation practice, Bates (38) summarizes the common assumption (in literature) of causal linkages between these four levels as follows: the four “… levels of criteria represent a causal chain such that positive reactions lead to greater learning, which produces greater transfer and subsequently more positive organizational results.” Bates further notes that several training evaluation studies and meta-analyses have failed to confirm such a linear causal pathway connecting training program inputs to outcomes through these four levels (38). Although the four levels of the Kirkpatrick and Kirkpatrick framework therefore cannot be assumed to represent an incremental four-level causal pathway, the framework provides possible sequential steps that an individual trainee might experience during and after training programs. It helps us by indicating where to look for contextual factors that could affect individual learning and its application within the participants’ organization.

One of the key components of the IPT of the implementers was the intent to bring about a *can-do* attitude among the participants. The implementers expected that mentoring by experienced public health professionals at the workplace of the participant would bring about such an attitudinal change. The initial program documents, however, did not elaborate on how the implementers expect such a change to take place in the individual participants. We had hypothesized that, in addition to organizational and environmental factors, such a positive organizational change could be linked to individual attributes of the participant, like the organizational commitment of the individual and the confidence that the individual places in his ability to produce such a change (15). The latter is related to perceived self-efficacy, identified as a mechanism that explains why some people feel *able* to take up some tasks while others do not, in spite of similar knowledge and skill levels (39). Similarly, organizational commitment and performance of an individual are closely related, as shown in several industrial and healthcare organizational settings (40–43).

In a local health system, where individual health managers work in small teams within organizations belonging to a broader network of healthcare institutions, the dynamic nature of the interactions at individual, team, institutional, and broader environmental levels contributes to whether participants apply what they learn and whether the expected organizational change manifests or not. Organizational frameworks therefore incorporate the role of such factors when analyzing healthcare organizational performance (29, 38). Workplace environment, nature of teams and teamwork, supervision received, attitude of state-level officials, and the needs and demands of the communities are all important factors that can affect organizational change after capacity-building. The various sub-units of a district health system, their interactions and influence on organizational performance are visualized in Figure 1.

Workplace environment in healthcare organizations has been identified as an important element explaining the application of learning from training programs in some settings, while not in others (44). In the conceptual framework of workplace learning proposed by Jacobs and Park, the inter-relationships between location of the training (learning occurs away from workplace), degree of planning (use of a systems approach), and an active role of the trainer were key variables in understanding workplace learning (45). Although not specific to healthcare organizations, this framework identifies important elements for developing the program theory of our intervention.

### Analysis of the context

**Figure 5:**
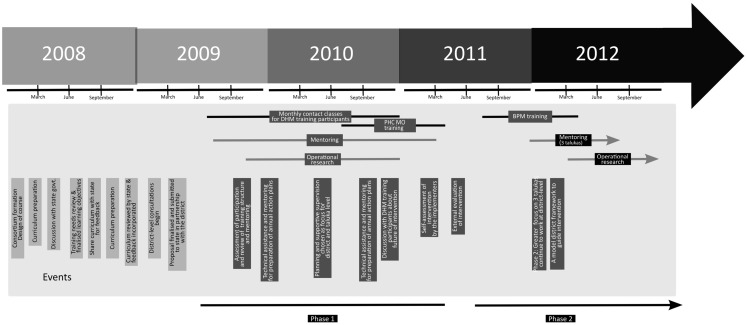
**Key events in course of the capacity-building intervention**.

While the theoretical frameworks provide plausible pathways through which the intervention inputs and outcome could be related, for the outcomes to effectively occur, local conditions matter. From a realist evaluation perspective, these are contextual factors that facilitate (or hinder) the outcome – they are crucial in refining the program theory. Contextual factors have been shown to influence organizational change in healthcare settings (46–52). While it is unlikely that each and every possible contextual factor will be identified, a documentary and literature review may help identify the most important and more obvious ones, especially if a plausible causal chain can be used as an anchoring point. We also used the mapping of key events (Figure 5) as a guide to identify relevant contextual factors.

We identified three key themes from the contextual analysis: the local effects of the ongoing decentralization of planning to district level in India, the acceptance and role of newly introduced non-medical program managers, and the local actors’ conceptualization of the district health services as a system. Below, we discuss these in more detail and analyze implications for our evaluation.

#### Pushes and pulls of a decentralizing bureaucracy

While the NRHM formally introduced decentralized planning in 2005, decentralization of the management of health services to the district level has been an *old* recommendation (53–56). Over the last decade, there has been an increasing trend of decentralization of planning and implementation of health care to the district level. There have also concurrently been calls for caution against hasty application of decentralization (sometimes characterized as a “disruptive innovation”) (57, 58), especially without creating an appropriate environment for decentralization to succeed. Studies stress the need for proper institutional capacity and an enabling environment before undertaking decentralization of health bureaucracies (7, 59–61).

The opportunity to conceive organizational change at their levels, through the design of their annual action plans, is an important contextual element for making sense of the response of Indian district health managers. Wherever health managers perceive this to be an opportunity, capacity-building programs may find fertile grounds and they can contribute to change planning practices. The available and perceived decision-spaces of health managers are another factor. For example, a recent study in Pakistan shows that perceived decision-space could vary from region to region, as well as among individuals within the same region (62). Parallel to the decentralization of the health bureaucracy is the ongoing process of decentralized governance at district and lower levels. Panchayati Raj Institutions (PRIs) are composed of elected representatives of the local governments at village, taluka, and district levels. The health services at the district level and below have been made accountable to the PRI, albeit they continue technical reporting to health officials at state level. The shifts in power dynamics in favor of representatives of PRI are important determinants of organizational change at district level in Karnataka. Capacity-building programs could work through providing health managers with the necessary capacity to negotiate with PRI members, and utilize their formal decision-spaces more effectively.

The relationship between decision-spaces available to health managers and their organizational commitment has been investigated. It has been shown that highly committed managers are able to bring about positive change through HRM interventions, even in settings where they have relatively constrained decision-spaces (43). Organizational commitment and decision-spaces available to health managers are important links in the pathway toward organizational change at the district level in health bureaucracies that are in the process of decentralizing.

#### Involvement of young management professionals in doctor-led teams

The NRHM, as explained above, introduced management professionals with a non-medical background into the health services at district and taluka levels. Their short-term contract appointments are in contrast to permanently tenured appointments of the doctor-health-managers in their team. These program managers were meant to strengthen planning and monitoring practices. However, their contribution to improving these processes is dependent on their relative position within existing health management teams, which remain led by doctors. The action of BPMs interested in making changes is determined not only by their technical capacity, but also their informal power vis-à-vis the doctors leading the traditional taluka teams. The same holds for DPMs at district level. The implementers’ initial assumption (as well as that of NRHM itself) on the role of program managers in enabling better planning and monitoring definitely needs to be examined in relation to existing team dynamics.

#### The district health services as a system

The implementers’ conception of a district health system as a complex system has guided the design and implementation of the intervention. Whether this approach resulted in creating truly functional teams of health managers depends on many individual and workplace factors. The implication of the team assumption on the performance of taluka and district participants therefore needs to be critically examined. In India, there is not much information on what a district health manager requires in terms of inputs, skills, and knowledge (63). Neither is there a well-established concept of a “district health team” among the health staff. Health managers may not perceive themselves as being part of a broader system that is supposed to work together in steering the district’s healthcare institutions toward improved performance. There are in fact both structural and functional problems in conceiving the Indian district health managers as functioning in teams.

**Figure 6:**
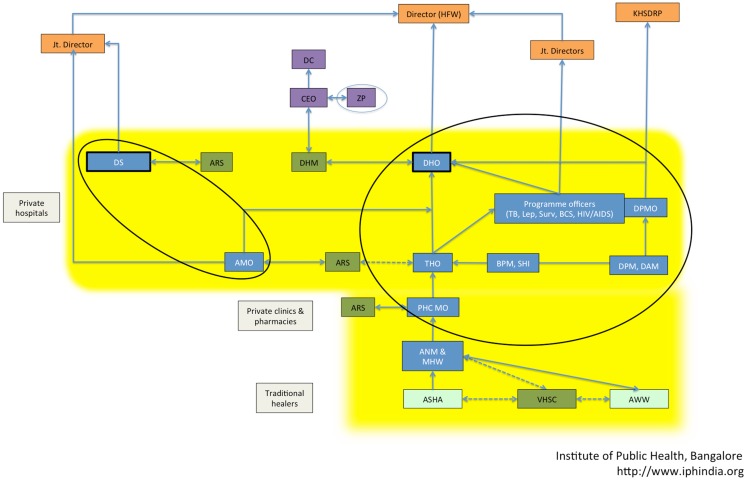
**Mapping of the actors in the Karnataka district health system showing their reporting relationships**. The various actors – state level officials (orange boxes), district administration (purple boxes), community participation platforms under NRHM (green boxes), health managers and health workers (blue boxes), other village-level health workers (light green), and private healthcare providers (gray boxes) – are shown. The actors targeted in the capacity-building intervention are circled. The yellow background indicates district health services. Abbreviations (in alphabetical order): administrative medical officer (AMO), Anganwadi worker (AWW), auxiliary nurse-midwife (ANM), Arogya Raksha Samiti (ARS – patient welfare committee), accredited social health activist (ASHA), block program manager (BPM), Chief Executive Officer (CEO), Deputy Commissioner (DC), district health mission (DHM), district health officer (DHO), District Surgeon (DS), health and family welfare (HFW), Karnataka Health Systems Development and Reforms Project (KHSDRP), male health worker (MHW), primary health center medical officer (PHC MO), Senior Health Inspector (SHI), Taluka Health Officer (THO), Village Health and Sanitation committee (VHSC), Zilla Panchayat (ZP – local self-government at the district level).

Structurally, the district health services are separated into a health and hospital wing. Both wings have independent reporting relationships to the state (see Figure 6). The health wing, in addition to management of smaller ( <100 bed) secondary hospitals and primary care facilities, oversees the operation of the many disease-control programs (e.g., vector-borne diseases and tuberculosis) and programs for reproductive and child health. Some of these programs have dual reporting lines: they report to the district health officer (DHO) as well as to dedicated disease-control program managers at the state level.

In the Tumkur capacity-building intervention, the implementers adopted a systems approach toward training health managers, on the assumption that they effectively worked with functional teams in their workplaces. This is evident in the selection of relatively diverse cadres of staff in the training program and in the team approach while training and mentoring. For example, mentoring visits targeted teams and not individual participants. The contact classes included DHO, program officers and hospital heads (all of them doctors), BPMs and DPMs, the administrative officers of the hospitals, and senior nurse-administrators at taluka and district levels. Although the implementers’ assumption was that the participants were members of a team of health managers, these teams were in fact not necessarily functional. Doctors are viewed as the health managers in charge, automatically sliding into and being accepted into positions of leadership and responsibility in their teams. New staffs are thus expected to enter into a reporting relationship with the doctors and are seen as subordinate in knowledge and in function. The factors related to the age gap, to the team relationships between medical and non-medical members of the teams, and to the relative power positions of members of the management teams all influence the functionality of the teams, and the degree to which non-medical team members will take up responsibilities.

### Putting it all together: The refined program theory and CMO configurations

Capacity-building of district health managers and its contribution to organizational change is influenced by relationships between actors and among the components of the district health system. In Figure 3, we represented the refined program theory of the intervention. As described above, we did this by critically examining the IPT and the assumptions of the implementers, in relation to existing literature on capacity-building and by drawing from a description of the most important aspects pertaining to the local context.

**Figure 7:**
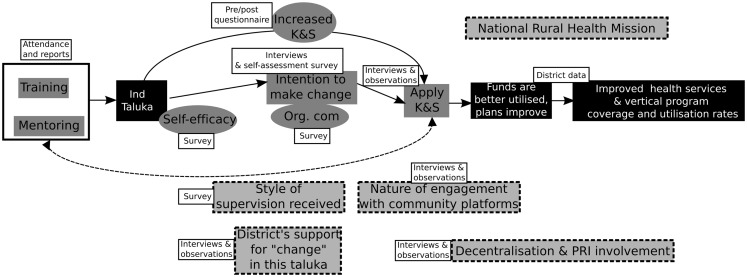
**Revised program theory based on incorporation of the implementer assumptions, theory (literature review), and analysis of the context**.

The program theory enables us to formulate a number of CMO configurations that can be subsequently used in guiding the analysis of data collected in course of the intervention (see Figure 7; Table 2). The CMO framework provides a lens through which to analyze empirical cases and build explanations for purposively chosen cases of positive and negative outcomes among the participants and teams. For example, the program theory points toward an important intermediate outcome, the intention to make positive organizational change after a training program. The contextual analysis and review of literature have also indicated important factors – individual mechanisms, institutional, and systemic factors (local context) – which could be mapped on a CMO framework. In this case, the CMO frame would start with positing possible contextual factors and mechanisms that could together bring about an intention to make positive change within a healthcare organization (outcome). We formulated three such CMO formulations based on the refined program theory. These formulations can be tested using a mix of qualitative and quantitative data to explain how positive organizational change can occur in response to such capacity-building programs (Table 2).

**Table 2:** **Table showing the progression from initial program assumptions toward explanatory mechanism, plausible contextual factors, and supporting theory, in relation to the expected outcome**.

Key IPT assumption	Supporting theory	Key contextual factor	Plausible mechanism identifiable from IPT and theory	Outcome of interest
Contact classes work through improving knowledge and/or skills, which are eventually applied. This results in improved performance	Outcomes of training programs accrue through four hierarchical levels: reaction (to training program), learning, behavior, and impact (37)	Team dynamics (nature of team and relationships) affects the individual with intention for positive change	Motivation of the participant toward positive organizational change – a “can-do” attitude in the IPT	Intention to make positive changes
		Socio-political environment in the taluka/district	
Mentoring participants at workplace facilitates application of knowledge and skills	Workplace environment in healthcare organizations has been identified as an important element that explains application of learning from training programs in some settings, while not in others (44)	Nature of supervision and district’s openness to “allow” change	Nature of commitment to organization	Identify/seek opportunities to make positive change in the organization’s performance
		Decentralized action plans and decision-making at district and lower levels. State and higher levels’ openness to change proposals	Self-efficacy	Improved annual action plans – better situation analysis, problem identification, allocation, and utilization of resources
A capacitated health manager can become an agent of positive organizational change	High commitment management literature shows the potential for change by committed staff in settings where resources could be mobilized (24)	Change proposals by districts are in line with state (or central) vision as well as address local needs (allocation and strategic alignment with external environment as per Sicotte et al.’s conceptual framework) (29)	Claiming and utilizing decision-spaces; organizational commitment and self-efficacy, in negotiating with superiors and community leaders	Taluka and districts plan improves. They identify more needs, mobilize more resources from state, and utilize it better (efficiency – both allocative and technical – improves)

## Discussion

### Program theory and realist evaluation

A program theory is a way of representing the expected relationships between the elements of an intervention implemented in a given context and its expected outcomes. Programs introduce new resources into a dynamic system. A program theory is a “set of propositions regarding what goes on in the black-box during the transformation of input into output; that is, how, via treatment inputs, a bad situation is transformed into a better one” (64, 65). A PT could be conceptualized as a logical and ordered description of the relationships between the various constitutive elements of an intervention, and the plausible pathways through which they interact with the elements of the system to produce the expected outcome. It draws upon the assumptions that the implementers have made in designing and implementing the intervention. It also incorporates the response of various actors within the system to the intervention and other contextual factors that could influence these actors and their responses to the intervention. A program theory is thus a pathway with interacting elements, showing how the inputs of an intervention could lead to the expected outcomes, taking into consideration contextual elements and the assumptions of the implementers on *how* they could achieve the objectives of the intervention.

In realist evaluation, Pawson and Tilley posit that programs are embedded in social systems. They stress the importance of understanding what works for whom, and under what conditions (66, 67). The realist evaluation approach focuses on the interaction between the mechanisms activated by the intervention and the context(s) in which it is implemented, specifically seeking to understand how this interaction in the various contexts produces changes that could lead to the outcomes (of interest to the evaluation). It is one of the several context-sensitive approaches to evaluate health programs at district level in low resource settings (23). In keeping with this, the program theory should identify intermediate steps in the pathway connecting the inputs of the intervention to the outcomes, the relationships between the steps and the conditions under which these occur. While existing theories provide plausible explanatory mechanisms through which inputs and outcomes could be related, the systemic factors unrelated to the intervention that could affect the outcome of the intervention (the context) are very important to understand how the intervention worked. Configurations of CMO based on program theory could be seen as plausible explanations of what worked for whom, and under what conditions. CMO configurations will enable us to collect data and test how the change occurred, in addition to whether the change occurred (or not). A critical reformulation of the IPT on the basis of empirical research, taking into consideration the conditions that could affect the outcome (like for instance other initiatives with similar outcomes and/or contextual conditions favorable or hindering the outcome locally) would eventually improve our understanding of the mechanisms of change, as well as enable a scientific evaluation.

### Implications for evaluation of district level HRM interventions

A district health system in India consists of a network of government-owned healthcare organizations providing primary, secondary, and tertiary care in addition to private hospitals and providers. A district or a taluka, ideally, would be the meeting point of top-down resource allocation and planning with bottom-up planning driven by local needs and people’s demands. Both dynamics would need to be integrated in a manner that ensures a well-performing health service that is responsive and provides good quality and equitable healthcare.

Local health systems at taluka and district level can be conceptualized as complex adaptive systems, a network of inter-related and inter-dependent organizations, which are relatively similar, yet dynamically interacting with each other and their environment. From a complex adaptive systems perspective, a linear causal logic model cannot be applied to evaluate a local health system intervention. Local contextual factors contribute to determine differences in outcomes, even if the appropriate resources for change are introduced (68). For example, the lack of a supportive learning and working environment in a given taluka hospital will be a barrier to realize the expected outcome, despite the introduction of new knowledge and skills through a training program, while it would have the potential to achieve this outcome in yet another hospital, where such an environment would exist.

### Analyzing local health system performance: The multipolar framework

**Figure 8:**
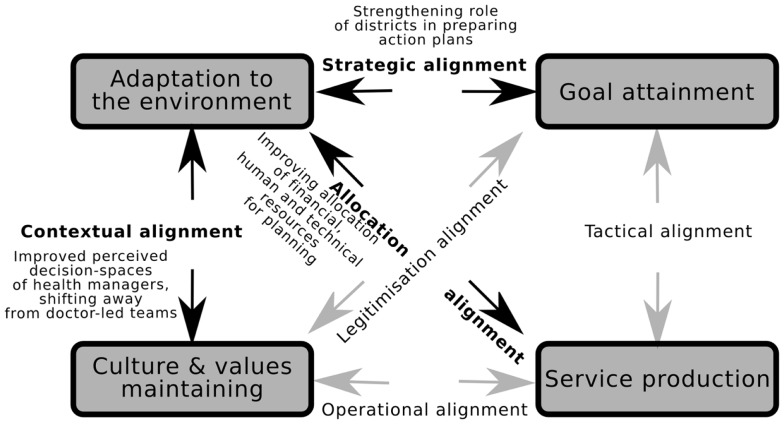
**Organizational change in Tumkur could be explained through identifying which of the alignments in the Multipolar framework were triggered by the intervention inputs**. The figure is based on the multipolar framework for assessing healthcare organization performance (28, 29).

A healthcare organization is a dynamic entity constantly interacting with a continuously changing environment through an internal dynamic exchange between its different functions. Based on a synthesis of several, often competing models of organizational performance, Sicotte et al. proposed a conceptual framework for analyzing performance in healthcare organizations. The overall performance of a healthcare organization is seen as being “determined by the dynamic equilibrium resulting from continual interaction of, and interchange among (these) four functions (attributes or properties of an organization)”: attaining the organization’s goals and adaptation to its external environment on one hand and its internal environment (the organizational culture and values) and its production (healthcare outputs) on the other (see Figure 8). This framework has been the basis for designing the multipolar performance framework, a heuristic that has been used to analyze performance of complex public sector healthcare organizations (30).

The pushes and pulls of the four *poles* result in six alignments, and the resulting performance of a given healthcare organization could be conceptualized as the net result of how the management team deals with the pushes and pulls along these various axes. For example, in the case of our program theory, change proposals introduced by a given *taluka* management team need to be in line with the district’s vision on one hand (allocation alignment) and the expectations of local actors (PRI members, community, and other actors at the taluka level) on the other (strategic alignment). In such a scenario, capacity-building interventions could operate through triggering changes in alignments, for example, by decreasing tensions in the allocation alignment through better negotiation skills provided to the taluka. The Tumkur capacity-building intervention implemented within the existing policy context of NRHM aimed to improve both allocation alignment (improved allocation of financial, human, and technical resources for planning) and strategic alignment (strengthening role of districts in making their own action plans). However, our program theory shows that in the present Indian district context, improved organizational performance would also need better contextual alignment (perceived decision-spaces of health managers and BPMs) and operational alignment (decreasing the social distance between doctors as leaders of health management teams and their other members, improved teamwork among the members of the team including the young program managers).

Programs for capacity-building of health workers operate at and across several levels – individual, institution, and system – with the positive outcomes, in this case, improved management of health services vary from one institution to another, and across different healthcare delivery teams. In a hospital where the doctor would be able to decrease the perceived social distance between herself and the BPM, there is likely to be a better operational alignment and higher chances of the BPM’s improved knowledge and skills manifesting as improved organizational performance. These plausible explanatory CMO configurations need to be tested using program data to understand which of the many possible organizational change mechanisms is operating in the Indian district setting.

## Conclusion

Capacity-building programs in health systems are an important HRM intervention, especially where adverse health outcomes are linked to poor management of health services. However, HRM studies in health and in other sectors have shown that training does not automatically generate positive organizational change. Efforts to understand the conditions under which capacity-building results in positive organizational change are limited by methodological difficulties, insufficient descriptions of the context under which interventions operated, and lack of attributability of changes observed to the intervention inputs. In this paper, we have described the process of building a revised program theory beginning from the IPT of the implementers, based on a thorough understanding of the local context and integrating relevant theoretical knowledge. This is helpful in understanding how, for whom, and under what conditions the intervention works. The resulting refined program theory clarifies the plausible causal links in the intervention, making it amenable for evaluation.

Although the design and delivery of HRM interventions could be standardized, the institutional (hospital or taluka/district) contexts and socio-political contexts vary from one institution/taluka to another and across districts. The plausible mechanisms through which a capacity-building program in health could bring about organizational change lie at the individual (self-efficacy and organizational commitment), teams (workplace characteristics), organization or district level (organizational characteristics of health services at the district level and the nature of the reporting relationships to levels above and below), and interaction with other talukas or healthcare organizations (local health systems). The Tumkur intervention has provided us with an opportunity to improve our understanding of these plausible mechanisms and their interactions with the context to produce a desirable outcome. The refined program theory can be used to further investigate how the capacity-building intervention worked, for whom it worked, and why.

## Conflict of Interest Statement

The first author was involved in the early implementation of the capacity-building intervention. All authors declare that there is no commercial or financial relationships that could be construed as a potential conflict of interest.

## Supplementary Material

The Supplementary Material for this article can be found online at http://www.frontiersin.org/Journal/10.3389/fpubh.2014.00089/abstract

## References

[1] van Olmen J Criel B Bhojani U Marchal B van Belle S Chenge MF The Health System Dynamics Framework: the introduction of an analytical model for health system analysis and its application to two case-studies. Health Cult Soc (2012) 2(1):1–12 10.5195/hcs.2012.71

[2] Tanner M Strengthening district health systems. Bull World Health Organ (2005) 83(6):403 10.1590/S0042-96862005000600003 15976886 PMC2626263

[3] World Health Organization. Working Together for Health: The World Health Report 2006. World Health Organization (2006).

[4] Cavalli A Bamba SI Traore MN Boelaert M Coulibaly Y Polman K Interactions between global health initiatives and country health systems: the case of a neglected tropical diseases control program in Mali. PLoS Negl Trop Dis (2010) 4(8):e798 10.1371/journal.pntd.0000798 20808908 PMC2923152

[5] Keugoung B Macq J Buvé A Meli J Criel B The interface between health systems and vertical programmes in francophone Africa: the managers’ perceptions. Trop Med Int Health (2011) 16(4):478–85 10.1111/j.1365-3156.2010.02716.x 21219552

[6] Biesma RG Brugha R Harmer A Walsh A Spicer N Walt G The effects of global health initiatives on country health systems: a review of the evidence from HIV/AIDS control. Health Policy Plan (2009) 24(4):239–52 10.1093/heapol/czp025 19491291 PMC2699244

[7] Dieleman M Gerretsen B van der Wilt GJ Human resource management interventions to improve health workers’ performance in low and middle income countries: a realist review. Health Res Policy Syst (2009) 7(1):7 10.1186/1478-4505-7-7 19374734 PMC2672945

[8] Adam T de Savigny D Systems thinking for strengthening health systems in LMICs: need for a paradigm shift. Health Policy Plan (2012) 27(Suppl 4):iv1–3 10.1093/heapol/czs084 23014149

[9] Sheikh K Gilson L Agyepong IA Hanson K Ssengooba F Bennett S Building the field of health policy and systems research: framing the questions. PLoS Med (2011) 8(8):e1001073 10.1371/journal.pmed.1001073 21857809 PMC3156683

[10] World Health Organization. Everybody’s Business – Strengthening Health Systems to Improve Health Outcomes. WHO’s Framework for Action. Geneva: World Health Organization (2007).

[11] Siddiqi K Newell J Robinson M Getting evidence into practice: what works in developing countries? Int J Qual Health Care (2005) 17(5):447–53 10.1093/intqhc/mzi051 15872024

[12] Goicolea I Coe AB Hurtig AK San Sebastian M Mechanisms for achieving adolescent-friendly services in Ecuador: a realist evaluation approach. Global Health Action (2012) 5(January):1–14 10.3402/gha.v5i0.18748 22855646 PMC3409349

[13] Wong G Greenhalgh T Westhorp G Buckingham J Pawson R RAMESES publication standards: realist syntheses. BMC Med (2013) 11(1):21 10.1186/1741-7015-11-21 23360677 PMC3558331

[14] Pawson R Greenhalgh T Harvey G Walshe K Realist review – a new method of systematic review designed for complex policy interventions. J Health Serv Res Policy (2005) 10(Suppl 1):21 10.1258/1355819054308530 16053581

[15] Prashanth NS Marchal B Hoeree T Devadasan N Macq J Kegels G How does capacity building of health managers work? A realist evaluation study protocol. BMJ Open (2012) 2(2):e000882 10.1136/bmjopen-2012-000882 22466036 PMC3330260

[16] Pawson R The Science of Evaluation: A Realist Manifesto. 1st ed London: Sage Publications (2013).

[17] Pawson R Tilley N Realistic evaluation bloodlines. Am J Eval (2001) 22(3):317–24 10.1177/109821400102200305

[18] Marchal B van Belle S van Olmen J Hoeree T Kegels G Is realist evaluation keeping its promise? A review of published empirical studies in the field of health systems research. Evaluation (2012) 18(2):192–212 10.1177/1356389012442444

[19] Chee G Pielemeier N Lion A Connor C Why differentiating between health system support and health system strengthening is needed. Int J Health Plann Manage (2013) 28(1):85–94 10.1002/hpm.2122 22777839 PMC3617455

[20] Bennett S Agyepong IA Sheikh K Hanson K Ssengooba F Gilson L Building the field of health policy and systems research: an agenda for action. PLoS Med (2011) 8(8):e1001081 10.1371/journal.pmed.1001081 21918641 PMC3168867

[21] Swanson RC Bongiovanni A Bradley E Murugan V Sundewall J Betigeri A Toward a consensus on guiding principles for health systems strengthening. PLoS Med (2010) 7(12):e1000385 10.1371/journal.pmed.1000385 21203584 PMC3006350

[22] Prashanth NS Marchal B Criel B Evaluating healthcare interventions: answering the “how” question. Indian Anthropol (2013) 43(1):35–50 Available from: http://www.mendeley.com/download/public/160002/6034808704/2e50d9be7b137c8126f34dacb27149c4008af420/dl.pdf23035767

[23] Svoronos T Mate KS Evaluating large-scale health programmes at a district level in resource-limited countries. Bull World Health Organ (2011) 89(11):831–7 10.2471/BLT.11.088138 22084529 PMC3209726

[24] Marchal B Dedzo M Kegels G A realist evaluation of the management of a well-performing regional hospital in Ghana. BMC Health Serv Res (2010) 10:24 10.1186/1472-6963-10-24 20100330 PMC2828434

[25] Kernick D The demise of linearity in managing health services: a call for post normal health care. J Health Serv Res Policy (2002) 7(2):121–4 10.1258/1355819021927782 11934378

[26] Lipsey MW Pollard JA Driving toward theory in program evaluation: more models to choose from. Eval Program Plann (1989) 12:317–28 10.1016/0149-7189(89)90048-7

[27] Connelly JB Evaluating complex public health interventions: theory, methods and scope of realist enquiry. J Eval Clin Pract (2007) 13(6):935–41 10.1111/j.1365-2753.2006.00790.x 18070265

[28] Marchal B Hoerée T da Silveira VC Van Belle S Prashanth NS Kegels G Building on the EGIPPS performance assessment: the multipolar framework as a heuristic to tackle the complexity of performance of public service oriented health care organisations. BMC Public Health (2014) 14(1):378 10.1186/1471-2458-14-378 24742181 PMC4020604

[29] Sicotte C Champagne F Contandriopoulos AP Barnsley J Béland F Leggat SG A conceptual framework for the analysis of health care organization’s performance. Health Serv Manage Res (1998) 11(1):24–48 10178368 10.1177/095148489801100106

[30] Marchal B Why Do Some Hospitals Perform Better than Others? A Realist Evaluation of the Role of Health Workforce Management in Well-Performing Health Care Organisations. Brussels: Faculty of Medicine and Pharmacy, Vrije Universiteit Brussel & Institute of Tropical Medicine, Antwerp (2011). Available from: www.itg.be/itgtool_v2/PersonalPages/PersonalPage.asp?Persnr=1665&L=E

[31] IPH Tumkur Team. Swasthya Karnataka Training in Tumkur. Bangalore: Institute of Public Health (2011). Available from: https://docs.google.com/file/d/0Bxp4UKSObSs9ODI5ZjYwNzktN2U4ZS00MTgyLTk0ZTAtZWQ2YTQzMTEyNzQ4/edit?hl=en_US

[32] Hoeree T Prasad V Jiang L Pongsupap Y External Evaluation of the Tumkur Training Course. Bangalore: Institute of Public Health (2012). Available from: https://docs.google.com/a/iphindia.org/file/d/0B-h6Rqii7VzEdTYyaWtkMjZMbWs/edit

[33] Government of India. National Rural Health Mission (2005-2012) Mission Document. New Delhi (2005). Available from: http://www.nird.org.in/Brgf/doc/Rural%20HealthMission_Document.pdf 16468284

[34] Rowe A Desavigny D Lanata C Victora C How can we achieve and maintain high-quality performance of health workers in low-resource settings? Lancet (2005) 366(9490):1026–35 10.1016/S0140-6736(05)67028-6 16168785

[35] Chopra M Munro S Lavis JN Vist G Bennett S Effects of policy options for human resources for health: an analysis of systematic reviews. Lancet (2008) 371(9613):668–74 10.1016/S0140-6736(08)60305-0 18295024

[36] Buchan J Human resources for health what difference does (“good”) HRM make? Hum Resour Health (2004) 2(6):1–7 10.1186/1478-4491-2-6 15182378 PMC425601

[37] Kirkpatrick DL Kirkpatrick JD Evaluating Training Programmes: The Four Levels. 3rd ed San Fransico, CA: Berrett-Koehler Publishers (2006).

[38] Bates R A critical analysis of evaluation practice: the Kirkpatrick model and the principle of beneficence. Eval Program Plann (2004) 27(3):341–7 10.1016/j.evalprogplan.2004.04.011

[39] Bandura A Self-efficacy mechanism in human agency. Am Psychol (1982) 37:122–47 10.1037/0003-066X.37.2.122

[40] Meyer JP Sampo VP Ian RG Richard DG Douglas NJ Organizational commitment and job performance: it’s the nature of the commitment that counts. J Appl Psychol (1989) 74(1):152–6 10.1037/0021-9010.74.1.152

[41] Maheshwari S Bhat R Saha S Commitment among state health officials & its implications for health sector reform: lessons from Gujarat. Indian J Med Res (2008) 127(2):148–53 18403792

[42] Mosadeghrad AM Ferlie E Rosenberg D A study of the relationship between job satisfaction, organizational commitment and turnover intention among hospital employees. Health Serv Manage Res (2008) 21(4):211–27 10.1258/hsmr.2007.007015 18957399

[43] Marchal B Dedzo M Kegels G Turning around an ailing district hospital: a realist evaluation of strategic changes at Ho Municipal Hospital (Ghana). BMC Public Health (2010) 10(1):787 10.1186/1471-2458-10-787 21184678 PMC3019197

[44] Clarke N Workplace learning environment and its relationship with learning outcomes in healthcare organizations. Hum Resource Dev Int (2005) 8(2):185–205 10.1080/1367886050010022817607137

[45] Jacobs RL Park Y A proposed conceptual framework of workplace learning: implications for theory development and research in human resource development. Hum Resource Dev Rev (2009) 8(2):133–50 10.1177/1534484309334269

[46] Mbindyo P Gilson L Blaauw D English M Contextual influences on health worker motivation in district hospitals in Kenya. Implement Sci (2009) 4(January):43 10.1186/1748-5908-4-43 19627590 PMC2727485

[47] Gray BH The influence of context on quality improvement success in health care: a systematic review of the literature. Milbank Q (2008) 86(4):529–32 10.1111/j.1468-0009.2010.00611.x 21166868 PMC3037175

[48] Squires JE Estabrooks CA Scott SD Cummings GG Hayduk L Kang SH The influence of organizational context on the use of research by nurses in Canadian pediatric hospitals. BMC Health Serv Res (2013) 13(1):351 10.1186/1472-6963-13-351 24034149 PMC3848566

[49] Victora CG Schellenberg JA Huicho L Amaral J El Arifeen S Pariyo G Context matters: interpreting impact findings in child survival evaluations. Health Policy Plan (2005) 20(Suppl 1):i18–31 10.1093/heapol/czi050 16306066

[50] Kapiriri L Martin DK Priority setting in developing countries health care institutions: the case of a Ugandan hospital. BMC Health Serv Res (2006) 6:127 10.1186/1472-6963-6-127 17026761 PMC1609114

[51] Byng R Norman I Redfern S Jones R Exposing the key functions of a complex intervention for shared care in mental health: case study of a process evaluation. BMC Health Serv Res (2008) 8(January):274 10.1186/1472-6963-8-274 19105823 PMC2627847

[52] Bigdeli M Zafar S Assad H Ghaffar A Health system barriers to access and use of magnesium sulfate for women with severe pre-eclampsia and eclampsia in Pakistan: evidence for policy and practice. PLoS One (2013) 8(3):e59158 10.1371/journal.pone.0059158 23555626 PMC3608621

[53] Mills A Vaughan JP Smith DL Tabibzadeh I Health System Decentralization: Concepts, Issues and Country Experience (1990). Available from: http://libdoc.who.int/publications/9241561378.pdf

[54] Segall M District health systems in a neoliberal world: a review of five key policy areas. Int J Health Plann Manage (2003) 18(Suppl 1):S5–26 10.1002/hpm.719 14661938

[55] Oliveira-Cruz V Hanson K Mills A Approaches to overcoming constraints to effective health service delivery: a review of the evidence. J Int Dev (2003) 15:41–65 10.1002/jid.965

[56] Gill K A Primary Evaluation of Service Delivery under the National Rural Health Mission (NRHM): Findings from a Study in Andhra Pradesh, Uttar Pradesh, Bihar and Rajasthan. Working Paper 1/2009. Planning Commission of India, New Delhi (2009). Available from: http://workspace.unpan.org/sites/internet/Documents/primary%20evaluation%20of%20service%20delivery.pdf

[57] Rae-Dupree J Disruptive Innovation, Applied to Health Care. The New York Times (2009). Available from: http://www.nytimes.com/2009/02/01/business/01unbox.html

[58] Tuli K The innovator’s prescription: a disruptive solution for health care. N Engl J Med (2009) 360(19):2038–39 10.1056/NEJMbkrev0810803

[59] World Health Organization. The World Health Report 2008: Primary Health Care Now More than Ever. Geneva: World Health Organization (2008).

[60] Bossert TJ Analyzing the decentralization of health systems in developing countries: decision space, innovation and performance. Soc Sci Med (1998) 47(10):1513–27 10.1016/S0277-9536(98)00234-2 9823047

[61] Fritzen SA Strategic management of the health workforce in developing countries: what have we learned? Hum Resour Health (2007) 5:4 10.1186/1478-4491-5-4 17319973 PMC1808474

[62] Bossert TJ Mitchell AD Health sector decentralization and local decision-making: decision space, institutional capacities and accountability in Pakistan. Soc Sci Med (2011) 72(1):39–48 10.1016/j.socscimed.2010.10.019 21134705

[63] Devadasan N Elias MA Training Needs Assessment for District Health Managers. Bangalore: Institute of Public Health (2008). Available from: http://www.iphindia.org; https://docs.google.com/file/d/0Bxp4UKSObSs9ODlhNjQwNjctMGNlNC00MjhlLWJmY2QtMGVmN2I3YmY2YWE2/edit?hl=en_US

[64] Astbury B Leeuw FL Unpacking black boxes: mechanisms and theory building in evaluation. Am J Eval (2010) 31(3):363–81 10.1177/1098214010371972

[65] Chen HT Issues in constructing program theory. New Dir Program Eval (2004) 1990(47):7–18 10.1002/ev.1551

[66] Pawson R Tilley N Realist evaluation. In: DPRN Thematic Meeting 2006 Report on Evaluation. Development Policy Review Network (2008). 35 p.

[67] Pawson R Sanjeev S Theory-driven evaluation of public health programmes. In: Killoran A Kelly MP, editors. Evidence-Based Public Health: Effectiveness and Efficiency. Oxford: Oxford University Press (2009). p. 41–62

[68] Mitleton-Kelly E Ten principles of complexity & enabling infrastructures. In: Mitleton-Kelly E, editor. Complex Systems and Evolutionary Perspectives on Organisations: The Application of Complexity Theory to Organisations. Amsterdam: Elsevier (2003). p. 23–50

