# Supplementary material for: Evaluation of Capacity-Building Program of District Health Managers in India: A Contextualized Theoretical Framework
Source: Front Public Health. 2014 Jul 25;2:89. doi: 10.3389/fpubh.2014.00089 (PMC4110717; doi:10.3389/fpubh.2014.00089)
Supplement: Supplementary file 1 [file DataSheet_1.ZIP › Data Sheet 1/File S1.DOCX]

**Supplementary file 1:** Description of the intervention

**Rationale and goal**: In 2000, a task force on health constituted by the government of Karnataka concluded that there was an overall neglect of public health principles and practice in planning, organization and management of health care services in the state^[[1]](#endnote-1)^. They also found the decision-making to be *ad hoc* and leadership qualities to be poor within the state health services. In their agenda for action, the task force recommended for strengthening public health management skills and competence. This reflects the situation commonly found at the taluka level health services in the state. A study undertaken by the designers of the intervention showed that there were many training programs organised to strengthen disease-control programs^[[2]](#endnote-2)^. However, a systemic approach to build management capacity among the doctors to manage taluka and district level healthcare institutions was lacking. In 2007, Institute of Public Health, Bangalore (IPH) conducted a Delphi study among senior government officials and public health professionals to understand the reason for poor health outcomes at the district level in India. One of the key findings of the study was the poor capacity to manage health services – planning and supervision of services - among the health managers at the taluka and district levels. The findings of the study are summarised below.


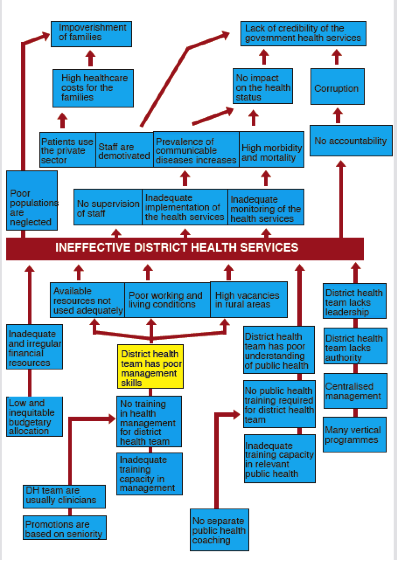


Figure 9: Findings from Delphi study on the issues for poor health outcomes in several Indian districts

Subsequently, IPH conducted a series of consultations and workshops with the government officials of the state health department and public health experts on ways to address this issue of poor management capacities. They found that the existing approach was centralised; it involved occasional and *ad hoc* posting of health managers to training programs at the state level, lasting from a few days to one week. IPH conducted a training needs assessment to understand the needs of the managers and conceived a blended training approach.

The capacity-building intervention at Tumkur was conceived in response to this situation and hence consisted of in-service training for district and taluka health managers, delivered through contact classes on a monthly basis at the district and follow-up visits by faculty (mentoring visits) to help the participants apply the knowledge/skills taught in their work and to understand problems or obstacles in applying them. The overall goal of the intervention as formulated in 2009 at the inception of the intervention was “…to improve service delivery and access to quality health care by strengthening district health services”. The implementers sought to do this by “building the capacity of the district health team so that this team is enabled to manage the health in the district in an effective and efficient manner”. See figure 10 for an overview of the structure of the intervention and the actors involved.

**Participants**: In figure 6 (of the paper), the participants of the intervention and their relationship to other actors in the district health system is shown. The implementers identified health managers (at taluka and district levels) and PHC doctors as participants in the intervention. It included the DHO and his team of program officers, THO and his immediate staff involved in management at taluka level and the heads of all the taluka hospitals as well as the district hospital along with their administrative and nursing heads. The newly created contractual cadre (as opposed to the others being permanent staff) of young program managers at the taluka (BPM) and district level (DPM) was also included.


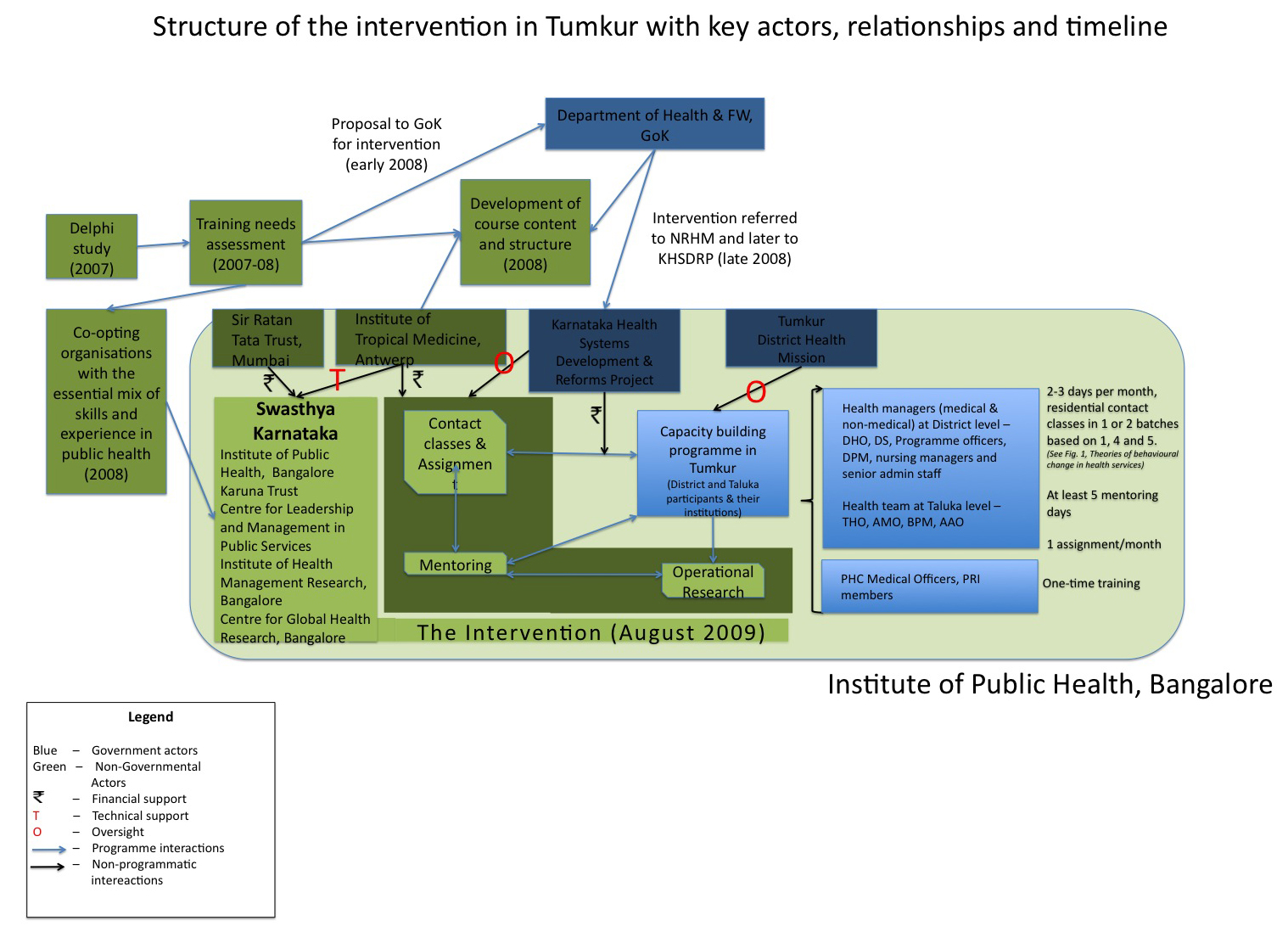


Figure 10: Structure of the Tumkur intervention and the actors involved

**Table 3**: Details of participants in the capacity-building intervention

| **Level** | **Designation** | **Num.** |
| --- | --- | --- |
| **District Health Office** | Health Officer (DHO)  Program Officer (DPO)  Program Manager (DPM)  Program Management Officer (DPMO)  Office Superintendent  Ayush Nursing Officer (NO) | 1  7  1  1  1  1 |
|  | **Total** | **12** |
| **District Hospital** | Dt. Surgeon  Administrator  RMO | 1  1  1 |
|  | **Total** | **3** |
|  | **Total District** | **15** |
| **Taluk Health Office** | Health Officer (THO)  Block program manager (BPM) | 10  10 |
|  | **Total** | **20** |
| **Taluk Hospital/ CHC** | Administrative Medical Officer (AMO)  Administrator | 13  13 |
|  | **Total** | **26** |
|  | **Total (from taluka)** | **46** |
|  | **Total (from health)** | **32** |
|  | **Total (from hospital/ CHC)** | **29** |
|  | **Overall total** | **61** |

**Implementation and actors**: IPH partnered with four other non-governmental organisations (a mix of development, training and research organisations from Karnataka) to form a consortium, *Swasthya Karnataka* (SK; *Kannada* for Healthy Karnataka). The reason for partnering with other organisations was to bring together a mix of skills related to public health management. The skill and experience mix of the organisations involved, as stated by them is provided in table 4. The Karnataka government through the Karnataka Health Systems Development and Reforms Project (KHSDRP) mandated SK to implement the intervention in Tumkur district. KHSDRP and the Tumkur district health services were oversaw the implementation of the program. SK raised funds to support the program development and implementation. KHSDRP funded the participation of the health managers in the contact classes for the Tumkur participants. The consortium partners shared the responsibility for organising the contact classes based on their expertise. While the formation of a consortium of organisations provided a wider complement of knowledge and skills on offer, there was a need for coordination among the partners, so that the intervention would be coherent across the contact classes and mentoring.

**Table 4**: Skills and experience of participating organisations in the intervention

| **Full Name** | **Specialty / Expertise** |
| --- | --- |
| Institute of Public Health, Bangalore | Research and training institute focussing on health systems |
| Karuna Trust | NGO working for health and development through community participation |
| Centre for Leadership and Management in Public Services | NGO working for improving management in public services |
| Centre for Global Health Research, Bangalore | Research centre in public health with special focus on health information systems |
| Institute of Health Management Research, Bangalore | Training Institute in health and hospital management |

**Elements of the intervention:** The intervention consisted of three components: training (contact classes), mentoring (follow-up visits to workplace of participants after contact classes) and operational research at district and taluka level. The main focus of the contact classes was the district and taluka health managers: health staff at district and taluka levels who are in charge of managing a health institution, service or a program. Faculty from the SK consortium visited participants’ workplaces after their contact classes. Each month, up to three mentoring visits were planned; each visit lasting up to one day. The program staff organised the mentoring visits to one of the participants’ workplaces and invited other participants (who were members of the same team) from that taluka to the place being visited by the mentor. For example, during a mentoring visit to Gubbi taluka hospital, the mentor would interact with all other intervention participants from Gubbi taluka, who would have been invited to participate in the visit on a mutually convenient day. The aim of the mentoring visits was to help participants complete learning assignments from their contact classes and to demonstrate the practical application of the content from the contact classes. The visits were also used to obtain feedback about the classes and to identify problems in applying the concepts discussed in the contact classes.

The contact class for the health managers was organised for 2-3 days every month, over a period of 17 months. Mentoring visits to their workplaces was conducted between the contact classes. The PHC medical officers were given a one time three-day training program, a condensed version of the health managers’ training program consisting only of topics relevant to PHC doctors. PRI representatives were invited to participate in a session on community participation for the health managers. The participants workplace problems (for example drug outages) were taken up as operational research topics by the implementers to better understand the problems faced by participants and use the lessons learnt in the contact classes and mentoring. The implementers saw this as a way of making the classes and the mentoring more relevant. The key elements of the intervention, actors involved and their relationship are shown in figure 9. This design of the intervention targeting different groups in the district was based on their objective to achieve *system-wide change.* Therefore all persons working at all levels in the system having managerial responsibilities were targeted for the training - from the district health authorities down to community members. Intensity of the training differed depending on the responsibilities of the persons. As such, three target groups were identified and for each group, the following interventions were given:

- Orientation: targeting community representatives; a short introduction;
- Capsule: 5-day course; an overview on health management, targeting personnel working at lower levels in the system: primary health care centres and sub-centres;
- Full course: 12 contact classes over a 1-year period with monthly mentoring visits at the workplace.

1. Government of Karnataka. (2001). Issues of Concern and an Agenda for Action, *Final report of the Task Force on Health and Family Welfare*. Bangalore: Task Force on Health and Family Welfare. [↑](#endnote-ref-1)
2. Devadasan, N., & Elias, M. A. (2008). *Training needs assessment for district health managers* (p. 78). Bangalore. [↑](#endnote-ref-2)
