# Supplementary material for: Evaluation of Capacity-Building Program of District Health Managers in India: A Contextualized Theoretical Framework
Source: Front Public Health. 2014 Jul 25;2:89. doi: 10.3389/fpubh.2014.00089 (PMC4110717; doi:10.3389/fpubh.2014.00089)
Supplement: Supplementary file 1 [file DataSheet_1.ZIP › Data Sheet 1/File S2.DOCX]

**Supplementary file 2: Organization and management of district health system in India**

Health services in a district include primary health centres, hospitals and other institutions that organise and manage health services across the district. In addition, there is a relatively large private health sector in India, consisting of a range of actors from single doctor clinics and traditional practitioners to large corporate hospitals. Although, they are a significant provider of curative healthcare services especially for secondary and tertiary care, the government health services are responsible for public health responsibilities (including primary health care) and provide health services to the poor. For the purpose of this paper, we will restrict the terms health services to the government health services, as the intervention we describe engaged only with the government health services.

The view of a district as an operational and actionable level in a health system is widely prevalent in public health literature^[[1]](#endnote-1),^^[[2]](#endnote-2)^. However, in India, districts are relatively large in terms of population. The mean population per district in Karnataka state in 2011 was 2.03 million, ranging from 554,762 people in the smallest Kodagu district to 95.88 million in the most populous Bangalore district^[[3]](#endnote-3)^. Many countries in Africa have much smaller districts; their population often ranges between 100,000 to 500,000. For example, the mean population per district in Ghana’s Western region district in 2010 was 139,766^[[4]](#endnote-4)^. In Karnataka state of southern India, there are 30 districts (see figure 10). Each district is further divided into administrative sub-units called *taluka.* The average population in a *taluka* is usually about a few hundred thousands. In Tumkur district of Karnataka, which has 10 talukas, taluka population in 2011 ranged from 168,039 in Koratagere *taluka* to 596,347 in Tumkur *taluka*. Furthermore, in Karnataka with the implementation of the *Panchayati raj* system of local self-governance, several public services administration such as health and education are devolved from the state level to the district level. Even though the health policy is often set by the national and state levels, in the decentralised system of governance, districts have the potential to play an important policy role in addition to being a management unit of the health system. In view of the relatively large population covered by the health services of a taluka, talukas will be considered as a local health system. The Indian talukas with a two tier health services system (a referral hospital administered by a hospital administrator and a network of PHCs with sub-centres administered by a Taluka health officer) are comparable in terms of their population to a typical district as described in public health literature.

In Karnataka, much like in most Indian districts, talukas have usually about 5-10 primary health centres (PHC) (figure 11 presents a schematic showing the organisation of health services in most Indian districts). The population coverage norm for a PHC is about 25,000 to 30,000, although this varies from region to region in the state. A cluster of four to five villages are provided with a subcentre, where an Auxiliary Nurse-Midwife (ANM) provides basic maternal and child health services, basic general health services and referral. About four to five subcentres are attached to each PHC. A PHC is the first point of contact between a doctor and the community; it has a team of medical and paramedical staff led by the doctor, who is also the administrator of the PHC. A hospital for secondary care is located at the *taluka* headquarters, usually the most populous town which is relatively better-connected with major cities in the region. In Tumkur district, there are 10 *talukas* with nine taluka hospitals (see figure 12). The district headquarters of Tumkur has a district hospital, which is supposed to provide tertiary care.

Among the states in southern India, Karnataka’s maternal mortality ratio estimate is at 178 maternal deaths for every 100,000 live births, higher than its neighbors, Kerala (81 per 100,000 live births) or Tamil Nadu (97 per 100,000 live births)(69). There is wide inter-district variation in health outcomes in India’s nearly 600 districts(70). Karnataka’s 30 districts vary widely with respect to important healthcare delivery indicators. For example in 2006, while immunisation coverage rate was 96% in Kodagu district, it was about 50% only in Raichur district(71). The Government of Karnataka identified wide disparities within districts when indicators were disaggregated further below to the taluka level(72). The Karnataka government classified nearly half of the 114 talukas as “backward” based on various development indicators (including health) are in the apparently better developed districts in Southern Karnataka(73). While the reasons for poor health outcomes in these talukas are likely to be multi-factorial, systemic failures due to poor planning and management of services is also directly contributory to poor healthcare in these areas (74,75).

**
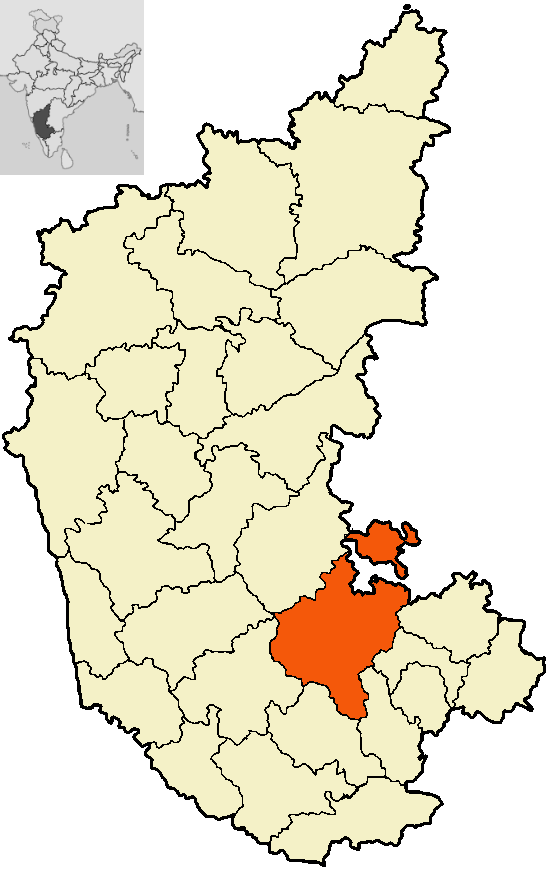
**

Figure 11: Map of Karnataka showing the 30 districts, of which Tumkur district is shaded orange

Karnataka, like most other states in India, does not train clinicians in management or in public health^[[5]](#endnote-5)^. Most of the doctors in managerial positions at districts and talukas are clinicians, who have been promoted to a public health position or as a hospital administrator. Public health or management training, if at all, is a result of individual pursuit or of *ad hoc* training programs organised by the state ranging from a few days to two weeks. An assessment of these programs found that they are perceived as “too theoretical and not useful”^[[6]](#endnote-6)^. Indeed, evaluation of externally funded, large-scale capacity-building programs in India have stressed the need for a systemic approach focusing on capacity-building as an institutional process that needs to sustain over time, stressing on moving beyond merely establishing new centralised training centres or training programs^[[7]](#endnote-7)^. A taluka health officer (THO) and a district health officer (DHO) are in charge of the management of the health services at the taluka and district level respectively. They are usually doctors with 10-20 years work experience in the health services. The district program officers work under the DHO in charge of disease-control programs or schemes implemented at the district level (typically, there are about eight such program officers for each district). A typical DHO has been a specialist doctor in one of the secondary or tertiary care hospitals in Karnataka. Similarly, a typical THO has been a PHC medical officer for several years or a specialist doctor with a post-graduation in a clinical specialty. Both cadres have no formal training in managing health services. In 2007, only two of the (then) 27 districts had a DHO with public health training, while a specialist clinician managed 14 district health services as a DHO^vi^.

The NRHM, that was implemented in 2005, incorporated decentralised planning and management of health services at the district level in its vision statement^[[8]](#endnote-8)^. As per NRHM guidelines, each district is the core unit of planning, budgeting and implementation of all the health programs. After NRHM implementation, the districts are expected to develop their own annual action plans called Program Implementation Plan (PIP). A District Health Society (DHS) was constituted with the DHO, the medical superintendent of the district hospital, and members from the district administration and elected representatives from the *Zilla Panchayat* (the local self-government at the district level). Similar bodies were instituted at all levels of the health services up to the PHC. The DHO is the administrative authority for health services at the district level, and is responsible for the personnel, funds and other resources allocated to health. He is the health action planner and manager of the health services in the district. The main administrative functions of the DHO include management of human, financial and material resources; planning and implementing health programs; providing leadership and motivating the staff; supervising, coordinating and controlling services at different levels and, responding to the changes in the external environment^vi^.

According to its mission document, NRHM sought to undertake an “architectural correction of the public health system to enable it to effectively absorb increased expenditure to provide accessible, affordable and accountable primary health care services to poor households in remote parts of rural India”. The mission document of NRHM echoes several international recommendations for strengthening primary health care and adopting a health systems approach. The translation of this into action is critically dependent on the management capacities of the district health team; a recent evaluation of NRHM has considered the question of capacity to plan and supervise health care at the district level and identified this as an important gap^[[9]](#endnote-9)^.

1. Segall, M. (2003). District health systems in a neoliberal world: a review of five key policy areas. *The International journal of health planning and management*, *18 Suppl 1*(October 2001), S5–26. [↑](#endnote-ref-1)
2. WHO. (2008). *The World Health Report 2008 : Primary health care now more than ever* (p. 148). Geneva. [↑](#endnote-ref-2)
3. Office of the Registrar General & Census Commissioner. (2011). Provisional Population Totals Paper 1 of 2011 India Series 1. New Delhi: Government of India. [↑](#endnote-ref-3)
4. Ghana Statistical Service. (2010). *2010 Population and Housing Census (PHC)*. Accra. [↑](#endnote-ref-4)
5. Sathyanarayan, T. N., & Babu, G. R. (2011). Creating a public health cadre in India: the development of a framework for interprofessional and inter-sector collaboration. *Journal of interprofessional care*, *25*(4), 308–10. doi:10.3109/13561820.2011.571354 [↑](#endnote-ref-5)
6. Devadasan, N., & Elias, M. A. (2008). *Training needs assessment for district health managers* (p. 78). Bangalore. [↑](#endnote-ref-6)
7. Potter, C., & Brough, R. (2004). Systemic capacity building: a hierarchy of needs. *Health Policy and Planning*, *19*(5), 336–345. doi:10.1093/heapol/czh038 [↑](#endnote-ref-7)
8. Government of India. (2005). *National Rural Health Mission (2005-2012) Mission Document* (p. 17). New Delhi. [↑](#endnote-ref-8)
9. National Rural Health Mission: **Fourth Common Review Mission Report**. 2010:130. [↑](#endnote-ref-9)
