# Supplementary material for: Evaluation of Capacity-Building Program of District Health Managers in India: A Contextualized Theoretical Framework
Source: Front Public Health. 2014 Jul 25;2:89. doi: 10.3389/fpubh.2014.00089 (PMC4110717; doi:10.3389/fpubh.2014.00089)
Supplement: Supplementary file 1 [file DataSheet_1.ZIP › Data Sheet 1/File S3.DOCX]

**Supplementary file 3: List of documents reviewed to understand the intervention**

| **Serial number** | **Title (number of documents)** | **Document type & publisher/source** | **Dated** |
| --- | --- | --- | --- |
| 1 | Strengthening district health management in Tumkur(1) | Government order by Government of Karnataka | 10 July 2008 |
| 2 | Mentoring notes (5) | Project document by SK/IPH | Multiple dates between August 2009 to August 2010 |
| 3 | Training needs assessment (DHM, BPM, financial management) (3) | Project document by SK/IPH | Multiple dates between August 2008 and August 2010 |
| 4 | Training reports (4) | Reports of contact classes: Project document by SK/IPH | Multiple dates from August 2009 to January 2011 |
| 5 | Curriculum development process documents (4) | Project document by SK/IPH | February 2009 |
| 6 | Tumkur Operational research reports (3) | Project document by SK/IPH | Multiple dates in 2009 & 2010 |
| 7 | Inauguration of training program (invitation and report) (1) | Project document by SK/IPH | August 2009 |
| 8 | SK consortium meeting minutes and notes (5) | Project document by SK/IPH | Multiple dates from April 2009 to January 2011 |
| 9 | Project proposal to Government and Sir Ratan Tata Trust (1) | Project document by SK/IPH | 2009 |
| 10 | Letters/communication to the Government by SK (4) | Project document by SK/IPH | 2009-2010 |
| 11 | NRHM PIP guidelines (2) | NRHM/Government of India | 2009 & 2010 |
| 12 | Tumkur district PIP (2) | Tumkur district | 2009 & 2010 |
| 13 | Government circulars and orders related to district health planning (4) | Government of India, Madhya Pradesh & Karnataka | 2009 & 2010 |
| 14 | Karnataka state PIP (1) | Government of Karnataka | 2009 |
| 15 | Evaluation reports (2) | Project document by SK/IPH | 2011 |
| 16 | Field visit reports and observation notes (8) | Project document by SK/IPH | 2009 & 2010 |

Total: 42 documents
