# Supplementary material for: Evaluation of Capacity-Building Program of District Health Managers in India: A Contextualized Theoretical Framework
Source: Front Public Health. 2014 Jul 25;2:89. doi: 10.3389/fpubh.2014.00089 (PMC4110717; doi:10.3389/fpubh.2014.00089)
Supplement: Supplementary file 1 [file DataSheet_1.ZIP › Data Sheet 1/File S4.PDF]

Interview guide

Greetings and introduce

Explanation about the research

Consent for recording the interview.

1) As a ______(Designation)_____, what is your role in the PIP?

Notes: *This question should ideally provide information on knowledge of the interviewee about the PIP process under NRHM. It should also reveal the interviewee's perceived involvement in the PIP. If interviewee suggests minimum role, ask whether he thinks he should be involved. What prevented him from involving.*

2) How was the PIP for this year for your district prepared?

Tags: Can you explain the whole process from the beginning?

Notes: This question is the key question of the interveiw, which is expected to capture the role played by the interviewee in this year's PIP. Details of when the process began, what obstacles were met and how s/he went about the process needs to be captured. Also, the interviewee's perceptions about who were involved in the PIP, and their roles should emerge.

Probes: When did you start (Probe for communication from directorate)?

Who was involved and what was the nature of involvement? Also, according to you, have everybody been involved to the extent needed?

(Probe specifically for PHCs, VHSCs, ANMs, ASHAs, Anganwadi workers and people from other departments – primary education, women and child development if they are left out by the interviewee)

How did you begin the process of making the plan? Who took the lead within the district to make the plan?

Tags: Meetings, orientation, other communication, emails. Outcomes of these.

What were the difficulties you faced in the process of making PIP(Probe for orientation on involvement)

Tags: time constraints, lack of consensus, poor understanding on process by some, role conflicts

How did you feel about the process of making the PIP this year?

What do you feel about the PIP?

3) Under NRHM according to guidelines, the district is supposed to involve communities right from village to the top administration in the district. What do you think of such a process?

Notes: This question is expected to capture the attitudes of the interviewee to bottom-up planning, his perceptions about the feasibility of such a process and encourage the participant to reflect on how such a process can be implemented, if at all. If interviewee agrees flatly to such a process, we need to discuss what s/he means by “participation” and “involvement”. What is the extent of involvement of communities that they expect, if at all they do see a role. The interviewer adopts a tone that questions the need for bottom-up planning to bring out the attitudes towards this.

Probes: Probe for feasibility in the district/area and attitudes towards involvement of various health staff and officials at all levels in planning in general.

Is it necessary to involve communities right from village level? Does this help in making an effective plan?

Can you suggest a better approach to planning at district/taluka/PHC/village/area level?
