# Supplementary material for: Evaluation of Capacity-Building Program of District Health Managers in India: A Contextualized Theoretical Framework
Source: Front Public Health. 2014 Jul 25;2:89. doi: 10.3389/fpubh.2014.00089 (PMC4110717; doi:10.3389/fpubh.2014.00089)
Supplement: Supplementary file 1 [file DataSheet_1.ZIP › Data Sheet 1/File S5.DOCX]

**Supplementary file 5: List of documents reviewed** (in alphabetical order)

**Organisational commitment** (34)

Allen, N. J., & Meyer, J. P. (1990). The measurement and antecedents of affective, continuance and normative commitment to the organization. *Journal of Occupational Psychology*, *63*(1), 1–18. doi:10.1111/j.2044-8325.1990.tb00506.x

Balabanova, D., Mckee, M., & Mills, A. (Eds.). (2011). *“Good health at low cost” 25 years on. What makes a successful health system*. London: London School of Hygiene & Tropical Medicine.

Banks, D. L. (2006). *Relationships between Organizational Commitment, Core Job Characteristics, and Organizational Citizenship Behaviors in United States Air Force Organizations*. Air University.

Berman, P. A., & Bossert, T. J. (2000). A Decade of Health Sector Reform in Developing Countries: What Have We Learned? In *DDM Symposium on Appraising a Decade of Health Sector Reform in Developing Countries* (pp. 0–20). Data for Decision Making Project, USAID.

Bowen, S., & Zwi, A. B. (2005). Pathways to “evidence-informed” policy and practice: a framework for action. *PLoS medicine*, *2*(7), e166. doi:10.1371/journal.pmed.0020166

Chirewa, B. (2012). Development of a practical toolkit using participatory action research to address health inequalities through NGOs in the UK: Challenges and lessons learned. *Perspectives in public health*, *132*(5), 228–34. doi:10.1177/1757913911399364

Chopra, M., Munro, S., Lavis, J. N., Vist, G., & Bennett, S. (2008). Effects of policy options for human resources for health: an analysis of systematic reviews. *Lancet*, *371*(9613), 668–74. doi:10.1016/S0140-6736(08)60305-0

Crisp, B. R. (2000). Four approaches to capacity building in health: consequences for measurement and accountability. *Health Promotion International*, *15*(2), 99–107. doi:10.1093/heapro/15.2.99

De Dreu, C. K. W., Evers, A., Beersma, B., Kluwer, E. S., & Nauta, A. (2001). A theory-based measure of conflict management strategies in the workplace. *Journal of Organizational Behavior*, *22*(6), 645–668. doi:10.1002/job.107

Dolea, C., Stormont, L., & McManus, J. (2010). *Increasing access to health workers in remote and rural areas through improved retention* (p. 72). Geneva: WHO, Geneva.

Fritzen, S. A. (2007). Strategic management of the health workforce in developing countries: what have we learned? *Human resources for health*, *5*, 4. doi:10.1186/1478-4491-5-4

Gautam, T., van Dick, R., & Wagner, U. (2001). Organizational Commitment in Nepalese Settings. *Asian Journal of Social Psychology*, *4*(3), 239–248. doi:10.1111/1467-839X.00088

Gray, B. H. (2008). The Influence of Context on Quality Improvement Success in Health Care: A Systematic Review of the Literature. *The Milbank quarterly*, *86*(4), 529–32. doi:10.1111/j.1468-0009.2008.00538.x

Greenhalgh, T, Robert, G., MacFarlane, F., Bate, P., & Kyriakidou, O. (2004). Diffusion of Innovations in Health Service Organisations: a systematic literature review. *The Milbank quarterly*, *82*(4), 581–629.

Greenhalgh, Trisha, Humphrey, C., Hughes, J., Macfarlane, F., Butler, C., & Pawson, R. (2009). How do you modernize a health service? A realist evaluation of whole-scale transformation in london. *The Milbank quarterly*, *87*(2), 391–416. doi:10.1111/j.1468-0009.2009.00562.x

Hackett, R. D., Bycio, P., & Hausdorf, P. A. (1994). Further assessments of Meyer and Allen’s (1991) three-component model of organizational commitment. *Journal of Applied Psychology*, *79*(1), 15–23. doi:10.1037/0021-9010.79.1.15

Henning, M. (2003). *Evaluation of the Conflict Resolution Questionnaire*. Auckland University of Technology.

Kumar, R., Ahmed, J., Shaikh, B. T., Hafeez, R., & Hafeez, A. (2013). Job satisfaction among public health professionals working in public sector: a cross sectional study from Pakistan. *Human resources for health*, *11*(1), 2. doi:10.1186/1478-4491-11-2

Louw, J. (1998). Programme evaluation: a structured assessment. In & T. S. J. Mouton, Muller, P., Franks (Ed.), *Theory and method in South African human sciences research: Advances and innovations* (pp. 255–268). Pretoria: HSRC.

Maheshwari, S., Bhat, R., & Saha, S. (2008). Commitment among state health officials & its implications for health sector reform: lessons from Gujarat. *The Indian journal of medical research*, *127*(2), 148–53.

Marchal, B., Dedzo, M., & Kegels, G. (2010a). Turning around an ailing district hospital: a realist evaluation of strategic changes at Ho Municipal Hospital (Ghana). *BMC public health*, *10*(1), 787. doi:10.1186/1471-2458-10-787

Marchal, B., Dedzo, M., & Kegels, G. (2010b). A realist evaluation of the management of a well-performing regional hospital in Ghana. *BMC health services research*, *10*(October 2000), 24. doi:10.1186/1472-6963-10-24

Mbindyo, P., Gilson, L., Blaauw, D., & English, M. (2009). Contextual influences on health worker motivation in district hospitals in Kenya. *Implementation science*, *4*, 43. doi:10.1186/1748-5908-4-43

Mbindyo, P. M., Blaauw, D., Gilson, L., & English, M. (2009). Developing a tool to measure health worker motivation in district hospitals in Kenya. *Human resources for health*, *7*, 40. doi:10.1186/1478-4491-7-40

Meyer, J. P., & Allen, N. J. (1991). A three-component conceptualization of organizational commitment. *Human Resource Management Review*, *1*(1), 61–89. doi:10.1016/1053-4822(91)90011-Z

Meyer, J. P., Paunonen, S. V., Gellatly, I. R., & Goffin, R. D. (1989). Organizational commitment and job performance: It’s the nature of the commitment that counts. *Journal of Applied Psychology*, *74*(1), 152–156. doi:10.1037/0021-9010.74.1.152

Mosadeghrad, A. M., Ferlie, E., & Rosenberg, D. (2008). A study of the relationship between job satisfaction, organizational commitment and turnover intention among hospital employees. *Health services management research an official journal of the Association of University Programs in Health Administration HSMC AUPHA*, *21*(4), 211–227.

O’Brien-Pallas, L., Birch, S., Baumann, A., & Murphy, G. T. (2001). Integrating workforce planning, human resources, and service planning. *Human Resources for Health Development Journal*, *5*(1-3), 2–16.

Pettigrew, A. M. (2012). Context and Action in the Transformation of the Firm: A Reprise. *Journal of Management Studies*, *49*(7), 1304–1328. doi:10.1111/j.1467-6486.2012.01054.x

Smither, J. W. (2005). Does Performance Improve Following Multisource Feedback? a Theoretical Model, Meta-Analysis, and Review of Empirical Findings. *Personnel Psychology*, *32*(1), 231–66. doi:10.1111/j.1744-6570.2005.514_1.x

Tayyab, S. (2007). An empirical assessment of organizational commitment measures. *Pakistan Journal of Psychological Research*, *22*(1-2), Sum 2007:1–21.

Tayyab, S., & Ajmal, M. (2006). *Antecedents and consequences of organizational commitment in Pakistan*. National Institute of Psychology, Quaid-i-Azam University, Islamabad.

Tsai, Y. (2011). Relationship between Organizational Culture, Leadership Behavior and Job Satisfaction. *BMC health services research*, *11*(1), 98. doi:10.1186/1472-6963-11-98

Yeboah-antwi, K., Snetro-plewman, G., Waltensperger, K. Z., Hamer, D. H., Kambikambi, C., Macleod, W., … Marsh, D. (2013). Measuring teamwork and taskwork of community-based “ teams ” delivering life-saving health interventions in rural Zambia : a qualitative study. *BMC Medical Research Methodology*, *13*(1), 1. doi:10.1186/1471-2288-13-84

**Self-efficacy** (19)

Arthur, W. J., Bennett, W. J., Edens, P. S., & Bell, S. T. (2003). Effectiveness of training in organizations: A meta-analysis of design and evaluation features. *Journal of Applied Psychology*, *88*(2), 234–245. doi:10.1037/0021-9010.88.2.234

Astbury, B., & Leeuw, F. L. (2010). Unpacking Black Boxes: Mechanisms and Theory Building in Evaluation. *American Journal of Evaluation*, *31*(3), 363–381. doi:10.1177/1098214010371972

Bandura, A. (1982). Self-efficacy mechanism in human agency. *American Psychologist*, *37*(2), 122–147. doi:10.1037/0003-066X.37.2.122

Bandura, A. (2006). Guide for constructing self-efficacy scales. In F. Pajares & T. C. Urdan (Eds.), *Self-Efficacy Beliefs of Adolescents* (pp. 307–337). Information Age Publishing.

Bombeke, K. (2012). *Patient-centredness in medical students : determinants and impact of communication skills training and clinical clerkships*. University of Antwerp.

Bradley, S., & Kamwendo, F. (2013). Human Resources for Health District health managers ’ perceptions of supervision in Malawi and Tanzania. doi:10.1186/1478-4491-11-43

Cheng, E. W. L. L., & Ho, D. C. K. K. (2001). A review of transfer of training studies in the past decade. *Personnel Review*, *30*(1), 102–118. doi:10.1108/00483480110380163

Gray, B. H. (2008). The Influence of Context on Quality Improvement Success in Health Care: A Systematic Review of the Literature. *The Milbank quarterly*, *86*(4), 529–32. doi:10.1111/j.1468-0009.2008.00538.x

Henning, M. (2003). *Evaluation of the Conflict Resolution Questionnaire*. Auckland University of Technology.

Hughes, B. B., Kuhn, R., Peterson, C. M., Rothman, D. S., & Solorzano, J. R. (2011). *Improving Global Health: Patterns of potential human progress*. *Health (San Francisco)* (Vol. 3).

Kaseje, D., Olayo, R., Musita, C., Oindo, C. O., Wafula, C., & Muga, R. (2010). Evidence-based dialogue with communities for district health systems’ performance improvement. *Global public health*, *5*(6), 595–610. doi:10.1080/17441690903418969

Luszczynska, A., Gutierrez-Dona, B., & Schwarzer, R. (2005). General self-efficacy in various domains of human functioning: Evidence from five countries. *International Journal of Psychology*, *40*(2), 80–89. doi:10.1080/00207590444000041

Luszczynska, A., Scholz, U., & Schwarzer, R. (2005). The general self-efficacy scale: multicultural validation studies. *The Journal of psychology*, *139*(5), 439–57. doi:10.3200/JRLP.139.5.439-457

Mathauer, I., & Imhoff, I. (2006). Health worker motivation in Africa: the role of non-financial incentives and human resource management tools. *Human resources for health*, *4*, 24. doi:10.1186/1478-4491-4-24

Munro, S., Lewin, S., Swart, T., & Volmink, J. (2007). A review of health behaviour theories: how useful are these for developing interventions to promote long-term medication adherence for TB and HIV/AIDS? *BMC public health*, *7*, 104. doi:10.1186/1471-2458-7-104

Pajares, F., & Urdan, T. C. (2005). *Self-efficacy Beliefs of Adolescents* (p. 380). Information Age Publishing.

Schwarzer, R., & Jerusalem, M. (1995). Generalized Self-Efficacy scale. In J. Weinman, S. Wright, & M. Johnston (Eds.), *Measures in health psychology: A user’s portfolio* (pp. 35–37). Windsor, England: NFER-NELSON.

Smither, J. W. (2005). Does Performance Improve Following Multisource Feedback? a Theoretical Model, Meta-Analysis, and Review of Empirical Findings. *Personnel Psychology*, *32*(1), 231–66. doi:10.1111/j.1744-6570.2005.514_1.x

Vachon, B., Désorcy, B., Camirand, M., Rodrigue, J., Quesnel, L., Guimond, C., … Grimshaw, J. (2013). Engaging primary care practitioners in quality improvement: making explicit the program theory of an interprofessional education intervention. *BMC health services research*, *13*(1), 106. doi:10.1186/1472-6963-13-106

**Workplace learning (12)**

Akbulut, Y., Esatoglu, a. E., & Yildirim, T. (2010). Managerial Roles of Physicians in the Turkish Healthcare System: Current Situation and Future Challenges. *Journal of Health Management*, *12*(4), 539–551. doi:10.1177/097206341001200408

Bombeke, K. (2012). *Patient-centredness in medical students : determinants and impact of communication skills training and clinical clerkships*. University of Antwerp.

Clarke, N. (2005). Workplace Learning Environment and its Relationship with Learning Outcomes in Healthcare Organizations. *Human Resource Development International*, *8*(2), 185–205. doi:10.1080/13678860500100228

Hager, P. (1997). Learning in the workplace. *Review of Research Monograph Series. Adelaide:  …*.

Jacobs, R. L., & Park, Y. (2009). A Proposed Conceptual Framework of Workplace Learning: Implications for Theory Development and Research in Human Resource Development. *Human Resource Development Review*, *8*(2), 133–150. doi:10.1177/1534484309334269

Leggat, S. G. (2007). Effective healthcare teams require effective team members: defining teamwork competencies. *BMC health services research*, *7*(1), 17. doi:10.1186/1472-6963-7-17

Mickan, S. M. (2005). Evaluating the effectiveness of health care teams. *Australian Health Review*, *29*(2), 211–217.

Nzinga, J., Ntoburi, S., Wagai, J., Mbindyo, P., Mbaabu, L., Migiro, S., … English, M. (2009). Implementation experience during an eighteen month intervention to improve paediatric and newborn care in Kenyan district hospitals. *Implementation science : IS*, *4*, 45. doi:10.1186/1748-5908-4-45

Oldham, G. R., & Cummings, A. (1996). Employee Creativity: Personal and Contextual Factors At Work. *Academy of Management Journal*, *39*(3), 607–634. doi:10.2307/256657

The Capacity Project. (2007). *Learning for Performance: A Guide and Toolkit for Health Worker Training and Education Programs* (p. 74). Chapel Hill: IntraHealth International.

Timmreck, T. C. (2001). Managing Motivation and Developing Job Satisfaction in the Health Care Work Environment. *Health Care Manager*, *20*(1), 42–58.

Vinokur-Kaplan, D., Jayaratne, S., & Chess, W. A. (1994). Job Satisfaction and Retention of Social Workers in Public Agencies, Non-profit Agencies, and Private Practice: The Impact of Workplace Conditions and Motivators. *Administration in Social Work*, *18*(3), 93–121.
